# Identifying TAD-like domains on single-cell Hi-C data by graph embedding and changepoint detection

**DOI:** 10.1093/bioinformatics/btae138

**Published:** 2024-03-06

**Authors:** Erhu Liu, Hongqiang Lyu, Yuan Liu, Laiyi Fu, Xiaoliang Cheng, Xiaoran Yin

**Affiliations:** School of Information and Control Engineering, Xi'an University of Architecture and Technology, Xi'an 710055, China; School of Automation Science and Engineering, Faculty of Electronic and Information Engineering, Xi’an Jiaotong University, Xi'an 710049, China; School of Automation Science and Engineering, Faculty of Electronic and Information Engineering, Xi’an Jiaotong University, Xi'an 710049, China; School of Automation Science and Engineering, Faculty of Electronic and Information Engineering, Xi’an Jiaotong University, Xi'an 710049, China; Department of Pharmacy, The First Affiliated Hospital of Xi’an Jiaotong University, Xi'an 710061, China; Department of Oncology, The Second Affiliated Hospital of Xi’an Jiaotong University, Xi'an 710004, China

## Abstract

**Motivation:**

Topologically associating domains (TADs) are fundamental building blocks of 3D genome. TAD-like domains in single cells are regarded as the underlying genesis of TADs discovered in bulk cells. Understanding the organization of TAD-like domains helps to get deeper insights into their regulatory functions. Unfortunately, it remains a challenge to identify TAD-like domains on single-cell Hi-C data due to its ultra-sparsity.

**Results:**

We propose scKTLD, an *in silico* tool for the identification of TAD-like domains on single-cell Hi-C data. It takes Hi-C contact matrix as the adjacency matrix for a graph, embeds the graph structures into a low-dimensional space with the help of sparse matrix factorization followed by spectral propagation, and the TAD-like domains can be identified using a kernel-based changepoint detection in the embedding space. The results tell that our scKTLD is superior to the other methods on the sparse contact matrices, including downsampled bulk Hi-C data as well as simulated and experimental single-cell Hi-C data. Besides, we demonstrated the conservation of TAD-like domain boundaries at single-cell level apart from heterogeneity within and across cell types, and found that the boundaries with higher frequency across single cells are more enriched for architectural proteins and chromatin marks, and they preferentially occur at TAD boundaries in bulk cells, especially at those with higher hierarchical levels.

**Availability and implementation:**

scKTLD is freely available at https://github.com/lhqxinghun/scKTLD.

## 1 Introduction

The chromatin architecture is nonrandom, which is organized with spatial structures of hierarchies, and plays an important role in multiple cellular processes, such as cell differentiation, gene regulation, epigenetic organization, and DNA replication (Dekker and Mirny 2016, Franke *et al.* 2016, Dekker *et al.* 2017, Marchal *et al.* 2019). High-throughput chromatin conformation capture (Hi-C) technology is devoted to the investigation of chromatin architecture by counting the interaction frequencies (IFs) between chromatin loci at a genome-wide scale (Liebermanaiden *et al.* 2009). In light of Hi-C, topologically associating domains (TADs) have been observed as structural blocks of chromatin loci and show a high degree of self-interacting (Dixon *et al.* 2012, Hou *et al.* 2012, Nora *et al.* 2012, Rao *et al.* 2014), and serve to confine genomic activity within their boundaries and restrict activity across their boundaries (Le Dily *et al.* 2014, Dixon *et al.* 2016). The boundaries of TADs are reported to be conserved across cell types and even species, demarcated by CTCF, housekeeping genes, cohesin complexes, and other histone marks (Dixon *et al.* 2012, Bonev and Cavalli 2016), and the disruption of TADs may result in gene misregulation and severe diseases, such as developmental disorders and cancers (Lupiáñez *et al.* 2015, Flavahan *et al.* 2016, Hnisz *et al.* 2016, Lupiáñez *et al.* 2016). Besides, further studies show that TADs are organized into a hierarchy with different structural levels (Weinreb and Raphael 2016), the more TADs move towards the inner of hierarchy, the higher their levels, and there is a more remarkable CTCF enrichment and gene expression near the boundaries of TADs with higher levels (An *et al.* 2019, Cresswell *et al.* 2020, Liu *et al.* 2022).

Getting off the ground, the exploration of TADs and their hierarchy are conducted mainly on Hi-C contact matrix of bulk cells, which are the mixture of thousands to millions of cells under different conditions. While entering the era of single-cell transcriptomics, the single-cell Hi-C technology allows the preparation of contact matrix of individual cells and the investigation of TAD-like domains on them, providing a means to get a deeper insight into these chromatin domains at single-cell level (Nagano *et al.* 2013, Nagano *et al.* 2017). For a short period of time, the low density of chromatin contacts in individual cells and the cell-to-cell variability led to a debate over whether TADs exist in single cells, until an imaging technology with kilo-base and nanometer-scale resolution was proposed to trace chromatin organizations, revealing the TAD-like structures at single-cell level (Bintu *et al.* 2018). Thus, an *in silico* method for identifying boundaries of TAD-like domains on single-cell Hi-C contact matrix seem necessary, although it remains a challenge to determine these boundaries due to the great sparsity and cell-to-cell variation of single-cell Hi-C data.

To our knowledge, several types of computational methods have been given out to identify TADs on bulk Hi-C data, such as (i) 1D statistic-based Directional index (DI) (Dixon *et al.* 2012), Insulation score (IS) (Crane *et al.* 2015), and TopDom (Shin *et al.* 2016); (ii) probabilistic model-based rGMAP (Yu *et al.* 2017), TADtree (Weinreb and Raphael 2016), and TADbit (Serra *et al.* 2017); (iii) graph-based Spectral (Chen *et al.* 2016), 3DNetMod (Norton *et al.* 2018), SuperTAD (Zhang *et al.* 2021), GRiNCH (Lee and Roy 2021), and deDoc (Li *et al.* 2018); as well as (iv) clustering-based ClusterTAD (Oluwadare and Cheng 2017), SpectralTAD (Cresswell *et al.* 2020), and TADpole (Solervila *et al.* 2020). Comparative analyses have shown that most of these methods can run on downsampled bulk Hi-C data (Dali and Blanchette 2017, Forcato *et al.* 2017, Zufferey *et al.* 2018, Li *et al.* 2020), but while these data are continuously downsampled to be as sparse as single-cell Hi-C data, almost all the methods become dysfunctional, demonstrating that they cannot be directly used on Hi-C data at single-cell level (Li *et al.* 2020). Currently, a few *ad-hoc* methods for identification of TAD-like domains on single-cell Hi-C data have been designed, including scHiCluster (Zhou *et al.* 2019), Higashi (Zhang *et al.* 2022), and deTOKI (Li *et al.* 2021). In the former two, a strategy of random walk and hypergraph representation learning is used to impute single-cell Hi-C contact matrix, and the TAD-like domains on it are called using TopDom and IS, respectively. In the last one, multiple rounds of nonnegative matrix factorization are carried out on single-cell Hi-C contact matrix, followed by a clustering to produce a consensus map, and the bins with minimal cluster rate (CR) are determined as the boundaries of TAD-like domains. These emerging methods seek out for TAD-like domains on ultra-sparse Hi-C contact matrix at single-cell level, but underutilize the global information far from the diagonal or have not concerned the running efficiency adequately, especially with the rapid increase in the number of single cells and the continuous improvement of resolution.

In this study, we propose scKTLD, an *in silico* method for identification of TAD-like domains on single-cell Hi-C data. It treats symmetric single-cell Hi-C contact matrix as an adjacency matrix for a graph, embeds the graph into a low-dimensional space with the help of sparse matrix factorization followed by spectral propagation, and identifies the TAD-like domains in the embedding space using a kernel-based changepoint detection. Beyond the existing methods, scKTLD addresses the following two issues. One is the combination of sparse matrix factorization and spectral propagation, so that the local smoothing and global community structures of the graph can be incorporated into the embeddings. The other is the detection of changepoints in the embedding space with a kernel model optimized by pruned exact linear time (PELT) (Killick *et al.* 2012), which allows the domain boundaries to be identified efficiently. The results tell that the embeddings by our scKTLD have an ability to reconstruct the Hi-C map with enhanced TAD-like structures, and the TAD-like domains can be identified more accurately and efficiently in most cases, compared with the other seven methods, including deTOKI, deDoc, scHiCluster, TopDom, SpectralTAD, GRiNCH, and Higashi, based on downsampled bulk Hi-C data as well as simulated and experimental single-cell Hi-C data. Moreover, with the help of scKTLD, it is observed that the TAD-like domain boundaries also exhibit conservation apart from heterogeneity within and across cell types in single cells, the bins identified as TAD-like domain boundaries with higher frequency may be more enriched for architectural proteins and chromatin marks, including CTCF, Rad21, Smc3, and H3K4me3, and the TAD-like domain boundaries in single cells preferentially occur at TAD boundaries in bulk cells, especially at those with higher hierarchical levels.

## 2 Materials and methods

### 2.1 Datasets

Experimental Hi-C data at bulk level as well as simulated and experimental Hi-C data at single-cell level are both involved in this article. For experimental bulk Hi-C data, 10 replicates of GM12878 and one replicate of K562 were derived from Rao’s bulk Hi-C experiment (Supplementary Table S1). In detail, the *.hic* files were downloaded from the Juicer data archive at https://bcm.app.box.com/v/aidenlab, and the contact matrices were extracted from these files via the dump command provided in juicer_tools (Durand *et al.* 2016). For simulated single-cell Hi-C data, there are two cell types being considered, with each type consisting of 100 single cells. In summary, a 3D physical model was established for each individual cell using the strategy proposed by Li *et al.* (2021) with the help of Integrative Modeling Platform (Bau and Marti-Renom 2012). Then for each model, a total of four single-cell Hi-C contact matrices were generated by weighted sampling of genomic loci at random, three of which are contact matrices prepared at different thresholds of contacting distance (500, 750, and 1000), and the leaving one is a reference contact matrix with ground-truth TAD-like domains (Supplementary Table S2). For experimental single-cell Hi-C data, the datasets for 17 GM12878 cells and 18 PBMC cells from Tan’s experiment (Tan *et al.* 2018) were downloaded at Gene Expression Omnibus (GEO) under accession number GSE80006 (Supplementary Table S3), and the datasets for three types of mouse cells, including 22 ZygMs, 18 ZygPs, and 70 Oocytes from Flyamer’s experiment were downloaded at GEO under accession number GSE117876 with the cells <50k contacts screened out (Supplementary Table S3). To make a fair comparison, the Hi-C data involved were binned to the same resolution for all the competing methods. Beyond the Hi-C data above, the ChIP-seq data for architectural proteins and histone marks, including CTCF, Rad21, Smc3, and H3K4me3, were also downloaded from ENCODE consortium (Dunham *et al.* 2012) to investigate the biological relevance of TAD-like domains (Supplementary Table S4). The downloaded ChIP-seq data were in bigWig format, and the program ‘bigWigAverageOverBed’ was used to segment the signals into bins of 10 kb for downstream processing and visualization.

### 2.2 Overview of scKTLD

**Figure 1.**
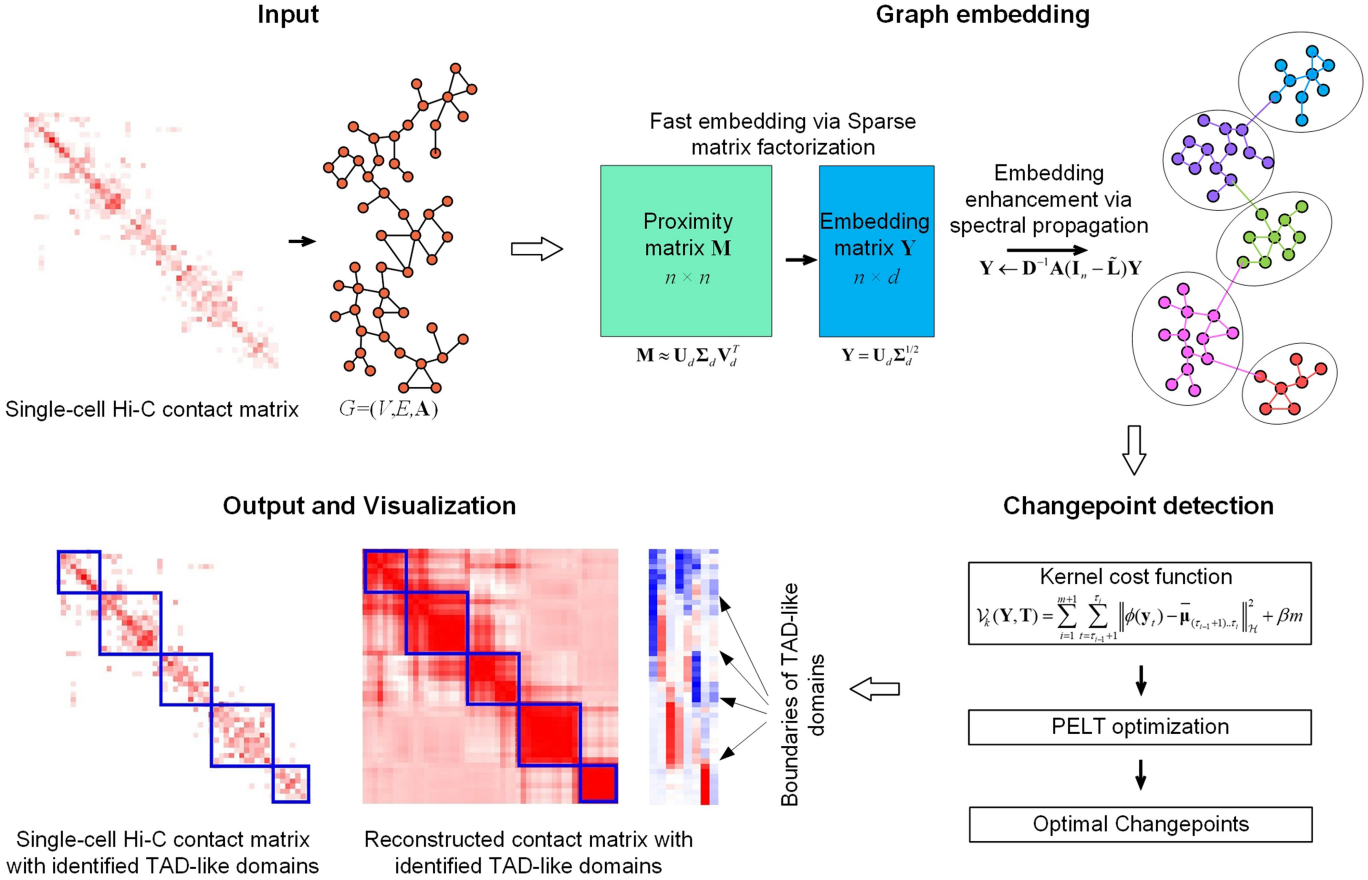
Overview of scKTLD. scKTLD takes a single-cell Hi-C contact matrix as input and outputs the TAD-like domains as well as the reconstructed contact matrix. It consists of three major steps. In the first step, scKTLD regards the contact matrix as the adjacency matrix for a weighted graph and embeds it into low-dimensional vectors via sparse matrix factorization. Then the vectors are further enhanced by propagation in the spectrally modulated network, trying to preserve the local smoothing and global community structures of the graph in the embedding space. In the second step, the changepoints in the embedding space are detected by minimizing a kernel cost function using PELT algorithm within a linear time consumption, and these changepoints are determined as the boundaries of TAD-like domains. Finally, the identified TAD-like domains are outlined on the heatmap of original single-cell Hi-C contact matrix or of contact matrix reconstructed with the embeddings according to the proximities between them.

Taking advantage of the natural graph-like attributes of single-cell Hi-C contact matrix, scKTLD treats TAD-like domain calling as a problem of changepoint detection in the embedding space from a weighted graph, in which each bin of contact matrix is treated as a node and the IFs between bins are the weights of corresponding edges. As shown in Fig. 1, scKTLD takes a single-cell Hi-C contact matrix as input, and outputs the identified TAD-like domains as well as a reconstructed contact matrix. It consists of three major steps. Step one, graph embedding. A symmetric single-cell Hi-C contact matrix is treated as the adjacency matrix of a weighted graph, the graph is then embedded into a low-dimensional space by combining a sparse matrix factorization with spectral propagation, while trying to preserve its graph properties, including the local smoothing and global community structures. Step two, changepoint detection. A dynamic programming-based algorithm called PELT is used to search for the optimal changepoints in the embedding space by minimizing a kernel cost function within a linear time consumption, and the changepoints are considered as the boundaries of TAD-like domains. Step three, results visualization. The identified TAD-like domains are outlined on the heatmap of input original single-cell Hi-C contact matrix or of reconstructed contact matrix in which the TAD-like domains are much clearer.

### 2.3 Graph embedding

#### 2.3.1 Sparse matrix factorization

Single-cell Hi-C contact matrix can be interpreted as an adjacency matrix for a weighted graph, and the sparse matrix factorization aims to obtain the initial distributional similarity-based embeddings of the graph in an efficient way (Zhang *et al.* 2019). Specifically, the graph defined by a contact matrix can be represented as *G* = (*V*, *E*, **A**), where *V* indicates node set, *E* denotes edge set, and **A** is adjacency matrix. Let *n* = |*V*| stand for the number of nodes, and **D** denote a diagonal degree matrix with $D_{ii}=\sum_{j}A_{ij}$. Then an *n*-by-*n* proximity matrix **M** can be constructed with each entry calculated as $r_{i}^{T}c_{j}$, where $r_{i}$ and $c_{j}$ refer to the embedding vectors of the node *v_i_* and context node *v_j_*, respectively. Besides, the negative samples are taken into account to avoid trivial solutions, thus the **M** can be defined as:

(1) $$M_{ij}=r_{i}^{T}c_{j}=\left\{ \begin{array}{ll} \ln p_{i,j}-\ln(\lambda P_{E,j}) & \text{if}\;(v_{i},v_{j})\in E\\ 0 & \text{if}\;(v_{i},v_{j})\notin E \end{array}\right.$$

where $p_{i,j}=A_{ij}/D_{ii}$, $P_{E,j}\propto {\left(\sum_{i|(i,j)\in E}p_{i,j}\right)}^{0.75}$ is the negative samples associated with context *v_j_*, and *λ* is a negative sample ratio. Subsequently, the proximity matrix **M** is factorized as $M\approx U_{d}\Sigma_{d}V_{d}^{T}$ using truncated singular value decomposition (tSVD), and the initial embeddings can be obtained via $Y=U_{d}\Sigma_{d}^{1/2}$, in which each row corresponds to a node. In order to improve the efficiency of matrix factorization, the factorization is performed via a sparse randomized tSVD (Halko *et al.* 2011). Compared to the traditional tSVD, it applies the random matrix theory to transform the tSVD of an *n*-by-*n* matrix **M** into the tSVD of a smaller *d*-by-*n* matrix **H **= **Q**^*T*^**M**, where **Q** is a matrix composed of *d* orthonormal columns, reducing the time complexity of matrix factorization from o(*n*^2^*d*) to o(*nd*^2^+|*E*|), where |*E*| is the number of edges in the whole graph. Taking a contact matrix for chromosome 1 of GM12878 cell (cell #1) in Tan’s dataset at 50 kb resolution as an example, The **M** is 4986 × 4986, while the **H** is only 128 × 4986, and the |*E*| is 62 888. We can see that *d* is much smaller than *n*, and the value of |*E*| is also small due to the ultra-sparsity of the single-cell Hi-C contact matrix, as a result, the sparse randomized tSVD will accelerate matrix factorization obviously.

#### 2.3.2 Spectral propagation

The initial embeddings above are enhanced by a propagation in a spectrally modulated network to incorporate the global and local structures of the graph. Assuming that the graph *G* has a normalized Laplacian matrix $L=I_{n}-D^{-1}A$, where $I_{n}$ is the identity matrix. The **L** can be decomposed as $L=U\Lambda U^{-1}$, where Λ is a diagonal matrix of eigenvalues, and **U** is a square matrix composed of the eigenvectors of **L**. Based on this, the propagation procedure can be described as follows:

(2) $$Y\leftarrow D^{-1}A(I_{n}-\tilde{L})Y$$

where $\tilde{L}=Ug(\Lambda )U^{-1}$ is a modulated Laplacian, with *g* indicating a spectral modulator, and $D^{-1}A(I_{n}-\tilde{L})$ is the resultant modulated network. The spectral modulator $g(\lambda )=e^{-\frac{1}{2}[{\left(\lambda -\mu \right)}^{2}-1]\theta }$ can be regarded as a band-pass filter to pass eigenvalues within a certain range and attenuate too large or too small ones, which will lead to the amplification of the global clustering and local smoothing according to high-order Cheeger’s inequality (Bandeira *et al.* 2013, Lee *et al.* 2014). Although the procedure provides a concise form to propagate the embeddings in the spectrally modulated network, the calculation of $\tilde{L}$ s still computationally expensive due to the explicit eigen-decomposition of **L** for large graphs. To circumvent this, a truncated Chebyshev expansion is used, so that $\tilde{L}$ can be approximated by an iterative polynomial operation (Supplementary Methods).

### 2.4 Changepoint detection

#### 2.4.1 Kernel cost function

Given a candidate segmentation of the sequential embeddings, a kernel cost function is used to estimate the sum of deviations from the piecewise mean in the reproducible kernel Hilbert space. The intuition behind the cost function is that the embeddings within the same TAD-like domain should have a similar distribution and be piecewise stationary in the transformed space. Let $Y=(y_{1},y_{2},\ldots ,y_{n}{)}^{T}$ stand for a clearer presentation of the sequential embeddings, and $T=(\tau_{0},\tau_{1},\ldots ,\tau_{m},\tau_{m+1})$ denote a set of *m* changepoints in order where $\tau_{0}$ and $\tau_{m+1}$ are artificially defined as 0 and *n*, respectively. Then the cost function $V_{k}(Y,T)$ can be given by:

(3) $$V_{k}(Y,T)=\sum_{i=1}^{m+1}\sum_{t=\tau_{i-1}+1}^{\tau_{i}}{\left\|\phi (y_{t})-{\bar{\mu }}_{(\tau_{i-1}+1)..\tau_{i}}\right\|}_{ℋ}^{2}+\beta m$$

where $\phi (y_{t})$ is a mapping function from ℝ^*d*^ to Hilbert space Η, which is implicitly defined as $\phi (y_{t})=k(y_{t},\cdot )$, and k(⋅,⋅) indicates the commonly used Gaussian kernel function, ${\bar{\mu }}_{(\tau_{i-1}+1)..\tau_{i}}\in ℋ$ is the empirical mean of the mapped segment $\left(\phi (y_{\tau_{i-1}+1}),\phi (y_{\tau_{i-1}+2}),\ldots ,\phi (y_{\tau_{i}})\right)$, ${\left\|\cdot \right\|}_{ℋ}$ denotes the norm in Η, and *β* is a penalty constant to control the trade-off between segmentation complexity and goodness-of-fit. Considering that it may be inconvenient to directly calculate $V_{k}(Y,T)$, the cost function can be simplified via the kernel trick during the calculation (Harchaoui and Cappé 2007):

(4) $$V_{k}(Y,T)=\sum_{i=1}^{m+1}\left[\sum_{t=\tau_{i-1}+1}^{\tau_{i}}k(y_{t},y_{t})-\frac{1}{\tau_{i}-\tau_{i-1}}\sum_{s,t=\tau_{i-1}+1}^{\tau_{i}}k(y_{s},y_{t})\right]+\beta m$$

#### 2.4.2 PELT optimization

The changepoints are determined by minimizing the cost function over possible numbers and locations with the help of a dynamic programming-based algorithm called PELT (Killick *et al.* 2012). This solution is chosen due to two main reasons. One is exactness. Unlike other approximate search methods, such as binary segmentation and bottom-up segmentation, PELT has an ability to find the exact global minimum of the cost function by recursively solving when the penalty is linear (Truong *et al.* 2020). The other is efficiency. This algorithm considers the observations sequentially, and uses an explicit pruning rule to discard some impossible locations from the potential changepoints in the next iteration, resulting in a significantly reduced computational cost that is linear in the number of data points (Supplementary Methods). These two advantages allow to find changepoints accurately and efficiently.

### 2.5 Reconstruction of contact Contact Matrix

To illustrate how well the local smoothing and global community structures, especially TAD-like structures, underlying contact matrix can be preserved or even enhanced by the graph embedding procedure in SCKTLD, a new artificial contact matrix is reconstructed with the embeddings produced by the graph embedding procedure. In detail, a proximity matrix **S** is first calculated, where $S_{ij}$ is the inner product between two embeddings for the *i*th and *j*th nodes in the graph. Then the values of elements in this proximity matrix are scaled to 0–1 using min–max normalization, and the normalized matrix is regarded as the reconstructed contact matrix.

### 2.6 Evaluation of TAD-like domains

Given two sets of TAD-like domains on an *n*-by-*n* Hi-C contact matrix, where *c* and *s* are the total numbers of domains in **U** and **V**, respectively,. Let,,$q_{j}=\left|V_{j}\right|/n$, and$r_{ij}=\left|U_{j}\cap V_{j}\right|/n$, in which |⋅| indicates the set size. The similarity between the two sets of TAD-like domains can be assessed via the following two metrics:.

#### 2.6.1 Adjusted mutual information

Mutual information (MI) is usually used to estimate how much information is shared between two variables, which can be defined as$\text{MI}(U,V)=\sum_{i=1}^{c}\sum_{j=1}^{s}r_{ij}\;\text{log}\;\frac{r_{ij}}{p_{i}q_{j}}$. As a variation, adjusted mutual information (AMI) can be used to evaluate the similarity between two partitions for a set. AMI is given by Li *et al.* (2021):

(5) $$\text{AMI}(U,V)=\frac{\text{MI}(U,V)-E\left\{\text{MI}(U,V)\right\}}{\max\left\{H(U),H(V)\right\}-E\left\{\text{MI}(U,V)\right\}}$$

where *H* denotes the Shannon entropy, and *E* denotes the mathematical expectation.

#### 2.6.2 Measure of concordance

A measure of concordance (MoC) is usually used to evaluate clustering assignments. To score the similarity between two sets of TAD-like domains, the bins within the same TAD-like domain are regarded as the elements within the same cluster; thus, the MoC between two sets of TAD-like domains can be defined as Zufferey *et al.* (2018):

(6) $$\text{MoC}(U,V)=\left\{ \begin{array}{ll} \;1 & \text{if}\;c=s=1\\ \frac{1}{\left(\sqrt{cs}-1\right)}\left(\sum_{i=1}^{c}\sum_{j=1}^{s}\frac{r_{ij}^{2}}{p_{i}q_{j}}-1\right) & \text{otherwise} \end{array}\right.$$

#### 2.6.3 TAD-adjR^2^

In addition to the similarity between TAD-like domains, another new metric named TAD-adjR^2^ has been proposed by An *et al.* (2019). With the help of this metric, the accuracy of domain assembly can be investigated by checking how much of the variation in the IFs of the Hi-C contact matrix can be explained by the classification of TAD-like domains. For each genomic distance, TAD-adjR^2^ is defined as follows:

(7) $${\hat{R}}_{\text{TAD}}^{2}=1-\frac{\frac{1}{b-p-1}\sum_{i=1}^{b}{\left(Y_{i}-{\hat{Y}}_{i}\right)}^{2}}{\frac{1}{b-1}\sum_{i=1}^{b}{\left(Y_{i}-\bar{Y}\right)}^{2}}$$

where *Y_i_* is the IFs for the *i*th bin, *b* indicates the number of bins that have the same genomic distance as this bin, and *p* denotes the number of so-called TAD-like domains whose sizes are no smaller than the distance. If the *i*th bin is within a TAD-like domain,${\hat{Y}}_{i}$ is the average IF within the TAD-like domain at a given genomic distance; otherwise,${\hat{Y}}_{i}$ indicates the average IF in the gap region at the given distance. And$\bar{Y}$ denotes the overall mean IF across all the bins at a given genomic distance.

### 2.7 Embedding and clustering of simulated cells

To investigate how well different types of simulated cells can be distinguished with the TAD-like domains called on them, these cells are embedded into a 2D space using a dimensionality reduction algorithm named multidimensional scaling (MDS) (Kruskal and Wish 1978), where the similarity between cells is scored by MoC and AMI between their TAD-like domains. In the implementation, MDS is carried out with the help of a function *MDS* in the Python package *sklearn.manifold*. Besides, to examine to what extent the embeddings can represent different types of cells in the embedding space, k-means clustering is performed on the embeddings of simulated cells, and the clustering results are evaluated via adjusted rand index (ARI).

### 2.8 Downsampling of bulk Hi-C data

To mimic the ultra-sparsity of single-cell Hi-C data, the replicates of bulk Hi-C contact matrices of GM12878 (Supplementary Table S1) are deeply downsampled with the help of the strategy proposed by Yardımcı *et al.* (2019), so that each replicate can be as sparse as single-cell Hi-C data. In the implementation, a downsampling ratio *R_d_* is first calculated as the number of target contacts over the total number of contacts for a given replicate, and the contact matrices for all chromosomes in this replicate are downsampled with this ratio. Then the contact matrix is converted into a set of pairwise individual interactions without considering the zero-valued elements, leaving a pairwise interaction vector of length *N*, where *N* is the sum of the individual elements of the contact matrix. Afterwards, a given number of interactions (N*R_d_) are randomly sampled from this vector without replacement using a uniform sampling procedure. Finally, the chosen interactions are re-binned into a new contact matrix with a fixed number of contacts. In this paper, each replicate is downsampled to contain only 0.350 million contacts, which is close to the median value of 0.339 million contacts per cell in Flyamer’s single-cell Hi-C experiment.

## 3 Results

### 3.1 Results on downsampled bulk Hi-C data at single-cell level

**Figure 2.**
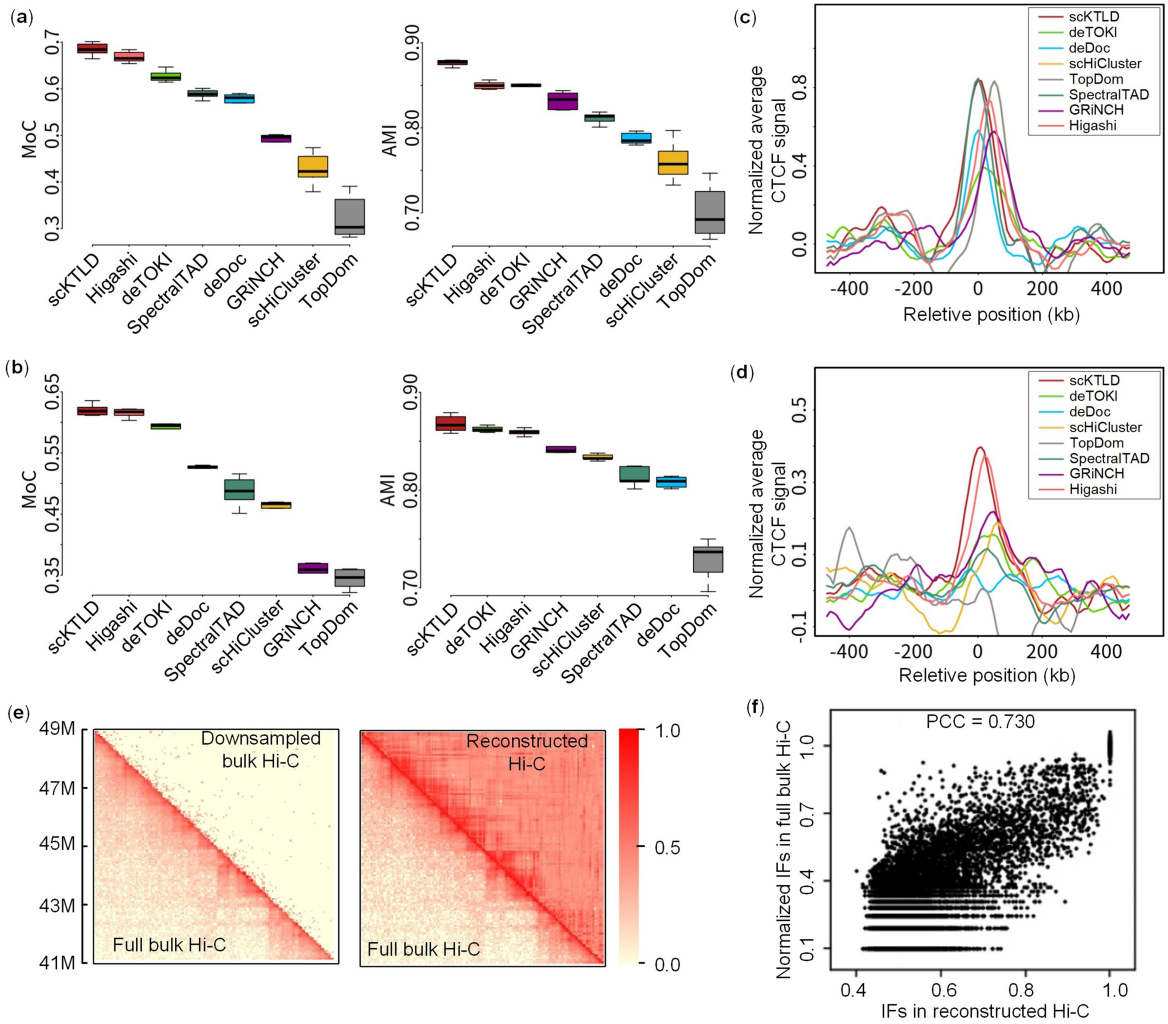
Results on downsampled bulk Hi-C data at the single-cell level. TADs were called by scKTLD and the other seven methods, including deTOKI, deDoc, scHiCluster, TopDom, SpectralTAD, GRiNCH, and Higashi, on both the full and deeply downsampled bulk Hi-C contact matrices for chromosome 1 of the GM12878 cell line from Rao’s bulk Hi-C experiment, and the similarity between these TADs in different cases was compared by means of MoC and AMI. (a) Similarity between the TADs called on bulk Hi-C data before and after downsampling at 50 kb resolution. (b) Similarity between the TADs called on the downsampled bulk Hi-C data across different resolutions (50 kb versus 25 kb). Beyond the two similarities, the enrichment of CTCF at TAD boundaries was also compared. (c) The normalized average CTCF signal per bin within 500 kb up-stream and down-stream flanking regions of each TAD boundary called on the full bulk Hi-C data. It should be noted that the curves for scHiCluster and TopDom are completely coincident since their TAD calling algorithm is exactly the same on the full bulk Hi-C data. (d) The normalized average CTCF signal per bin within 500 kb up-stream and down-stream flanking regions of each TAD boundary is called on the downsampled bulk Hi-C data. In addition, the superiority of our scKTLD derived from the graph embedding step while dealing with ultra-sparse Hi-C data was illustrated. (e) The left panel shows an intuitive comparison between the full and downsampled bulk Hi-C contact matrices (GSM1551550_HIC001, 41M–49M) at 50 kb resolution, while the right panel is a comparison between the full bulk Hi-C contact matrix and the corresponding reconstructed contact matrix. The IFs in these matrices are scaled to 0–1 using min–max normalization. (f) The scatter plot of the IFs in the full bulk Hi-C contact matrix versus those in the reconstructed contact matrix. PCC refers to the Pearson correlation coefficient.

To investigate the ability of scKTLD in identifying TAD-like domains on ultra-sparse single-cell Hi-C data, the contact matrices for the GM12878 cell line from Rao’s bulk Hi-C experiment (Rao *et al.* 2014) were deeply downsampled, so that each replicate only contains 0.350M contacts, which is close to the median value of 0.339M contacts per cell in Flyamer’s single-cell Hi-C experiment. Then, on both the full and downsampled bulk Hi-C data, scKTLD was compared with the other seven TAD callers, including deTOKI, deDoc, scHiCluster, TopDom, SpectralTAD, GRiNCH, and Higashi. It is worth noting that among these methods, deTOKI, scHiCluster, and Higashi are devoted to the identification of TAD-like domains on single-cell Hi-C data, while the remaining four are designed for bulk Hi-C data and are reported to be applicable to lower sequencing depths (Cresswell *et al.* 2020, Lee and Roy 2021, Liu *et al.* 2022). This comparison was conducted in the following three ways: Firstly, similarity between the TADs was called for on bulk Hi-C data before and after downsampling. The similarity is quantified with MoC and AMI by treating the bins in a TAD as the elements in a cluster or as the subset in a partition. It is obvious that scKTLD can reach both the highest MoC and AMI (median MoC = 0.69 and median AMI = 0.88) (Fig. 2a). The results are also supported by the visualized TADs marked on the heatmaps of contact matrices (Supplementary Fig. S1). Secondly, similarity between the TADs called on downsampled bulk Hi-C data across different resolutions. In this case, scKTLD still has the highest similarity (median MoC = 0.63 and median AMI = 0.87) (Fig. 2b), followed by Higashi and deTOKI, indicating that the TADs called by the three methods show higher consistency across resolutions. Finally, the enrichment of architectural proteins and histone marks, including CTCF, Rad21, Smc3, and H3K4me3,. The average ChIP-seq signals per bin within 500 kb up-stream and down-stream flanking regions of each TAD boundary were calculated and normalized by subtracting the average background signals in the up-stream −500 kb to −100 kb and the down-stream 100–500 kb regions. As shown in Fig. 2c, an enrichment of CTCF can be observed for all the methods on the full bulk Hi-C data, with scKTLD, SpectralTAD, and TopDom having the higher peaks. However, once running on the downsampled bulk Hi-C data, the results change greatly for most methods (Fig. 2d). The enrichment decreases dramatically for deTOKI and SpectralTAD, especially for deDoc and TopDom, leaving scKTLD having the most apparent CTCF peak at the boundaries called on the downsampled bulk Hi-C data. The enrichments of Rad21, Smc3, and H3K4me3 for these methods are also given in Supplementary Figs S2 and S3, and the comparison results tell that scKTLD is still competitive, especially on the downsampled bulk Hi-C data at single-cell level.

It is believed that the superiority of our scKTLD while dealing with downsampled bulk Hi-C data is mainly derived from its graph embedding step. As shown in the left panel of Fig. 2e, the TAD structures near the diagonal of the downsampled bulk Hi-C contact matrix are much more difficult to distinguish compared with those of the full bulk Hi-C contact matrix, which may frustrate the other methods that only make use of the local features near the diagonal. Different from these methods, scKTLD seeks to find TADs in the embedding space where the structures of the whole graph can be preserved. To clarify this point further, a new contact matrix was reconstructed with the embeddings given by our graph embedding step on the downsampled bulk Hi-C data, and a comparison between the full bulk Hi-C contact matrix and the corresponding reconstructed contact matrix was made (Fig. 2e, right panel). As we can see, the TAD structures in the reconstructed contact matrix, especially near the diagonal, show a considerable similarity with those in the original bulk Hi-C contact matrix, and the IFs in the reconstructed contact matrix are also highly correlated with those in the full bulk Hi-C contact matrix (PCC = 0.73, Fig. 2f). These indicate that our graph embedding step has the ability to capture and enhance the key block structures in the ultra-sparse Hi-C data.

### 3.2 Results on simulated single-cell Hi-C data

**Figure 3.**
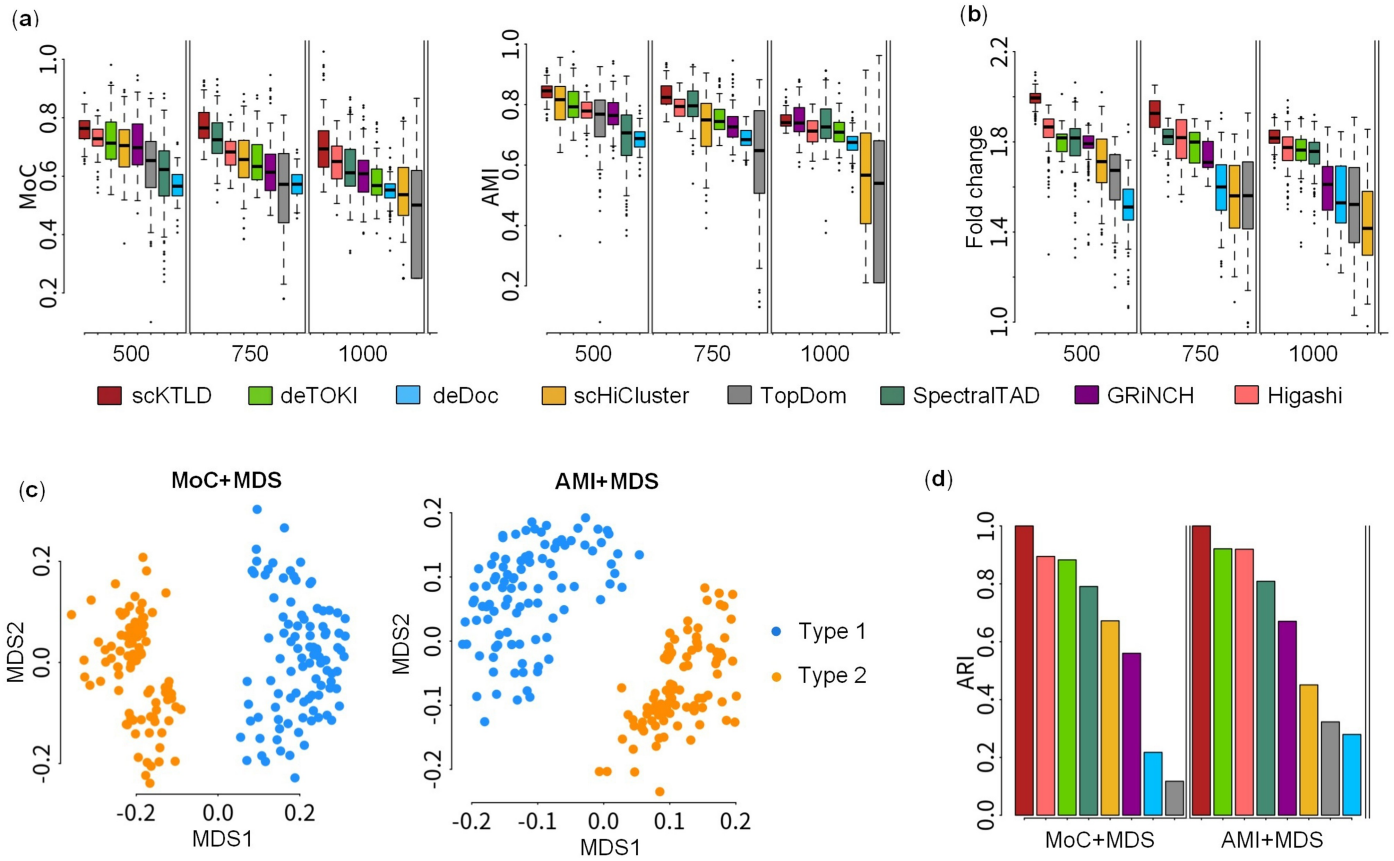
Results on simulated single-cell Hi-C data. TAD-like domains were called by scKTLD and the other seven methods, including deTOKI, deDoc, scHiCluster, TopDom, SpectralTAD, GRiNCH, and Higashi, on two types of simulated single-cell Hi-C contact matrices at 50 kb resolution, and the performance of these methods was compared in terms of accuracy and compactness of the called TAD-like domains. (a) Accuracy of TAD-like domains, which is scored by the similarity between the TAD-like domains called on simulated single-cell Hi-C data and those preset on reference Hi-C data. (b) Compactness of TAD-like domains, which is scored by the fold change of the average spatial distance between loci in adjacent TAD-like domains to that within TAD-like domains. To investigate the specificity of the TAD-like domains across cells, the TAD-like domains were called on the two types of simulated single-cell Hi-C data at the distance threshold of 750, and the cells were then embedded by MDS, where the MoC and AMI between the TAD-like domains called on these cells were used to score the similarity between cells. (c) Scatter plot of simulated single cells in the embedding space. Besides, these cells were further clustered in the embedding space using k-means, and the ARI for different methods was compared. (d) Barplot of ARI for the clusters of the two types of cells.

The simulated single-cell Hi-C data for two cell types were prepared with the help of the strategy proposed by Li *et al.* (Supplementary Methods) (Li *et al.,* 2021). In the preparation, the thresholds of contacting distance were separately set to three different values (500, 750, and 1000) to simulate multiple different experimental conditions. These simulated data were fed into our SCKTLD and the other seven methods to investigate their performance. Firstly, the accuracy of the TAD-like domains. The so-called TAD-like domains were compared with ground-truth TADs using MoC and AMI to examine their accuracy. As shown in Fig. 3a, the median MoC and AMI for scKTLD are the highest at most distance thresholds. It suggests that scKTLD can identify TAD-like domains more accurately on the simulated single-cell Hi-C data in most cases. Secondly, the compactness of the so-called TAD-like domains. TADs are observed as structural blocks of chromatin loci and show a high degree of self-interacting; that is to say, the loci pairs within domains are closer in distance than those outside. In light of that, the compactness of TAD-like domains has been taken into account by the 3D physical models in the simulation. Thus, the fold change of the average spatial distance between loci in adjacent TAD-like domains to that within TAD-like domains was calculated (Fig. 3b). As expected, our scKTLD always achieves the highest fold change regardless of distance thresholds, indicating that the TAD-like domains called by our method are more compact in space. Besides, the TAD-like domains given by scKTLD are marked on the heatmaps of simulated single-cell Hi-C contact matrices, and it seems that these domains are generally in line with the visualized 3D physical model for the corresponding chromosome regions in the simulation (Supplementary Fig. S4). Finally, the specificity of the so-called TAD-like domains across cells. Considering that the chromatin structures across simulated cells, especially across two different types of simulated cells, are different, we wondered the specificity of the TAD-like domains called on individual cells and whether or to what extent the two types of cells could be distinguished with these TAD-like domains. Thus, the simulated cells were embedded in MDS, where the MoC and AMI between TAD-like domains were used to score the similarity between cells (Fig. 3c and Supplementary Fig. S5). As expected, the two types of simulated cells can be separated in the embedding space with the TAD-like domains called on them, and the separation appears clearer with those called by scKTLD. Furthermore, the embeddings were clustered using k-means (Fig. 3d), and as we can see, scKTLD can achieve the highest ARI. It shows that the simulated single cells can be better distinguished with the TAD-like domains called scKTLD.

### 3.3 Results on experimental single-cell Hi-C data

**Figure 4.**
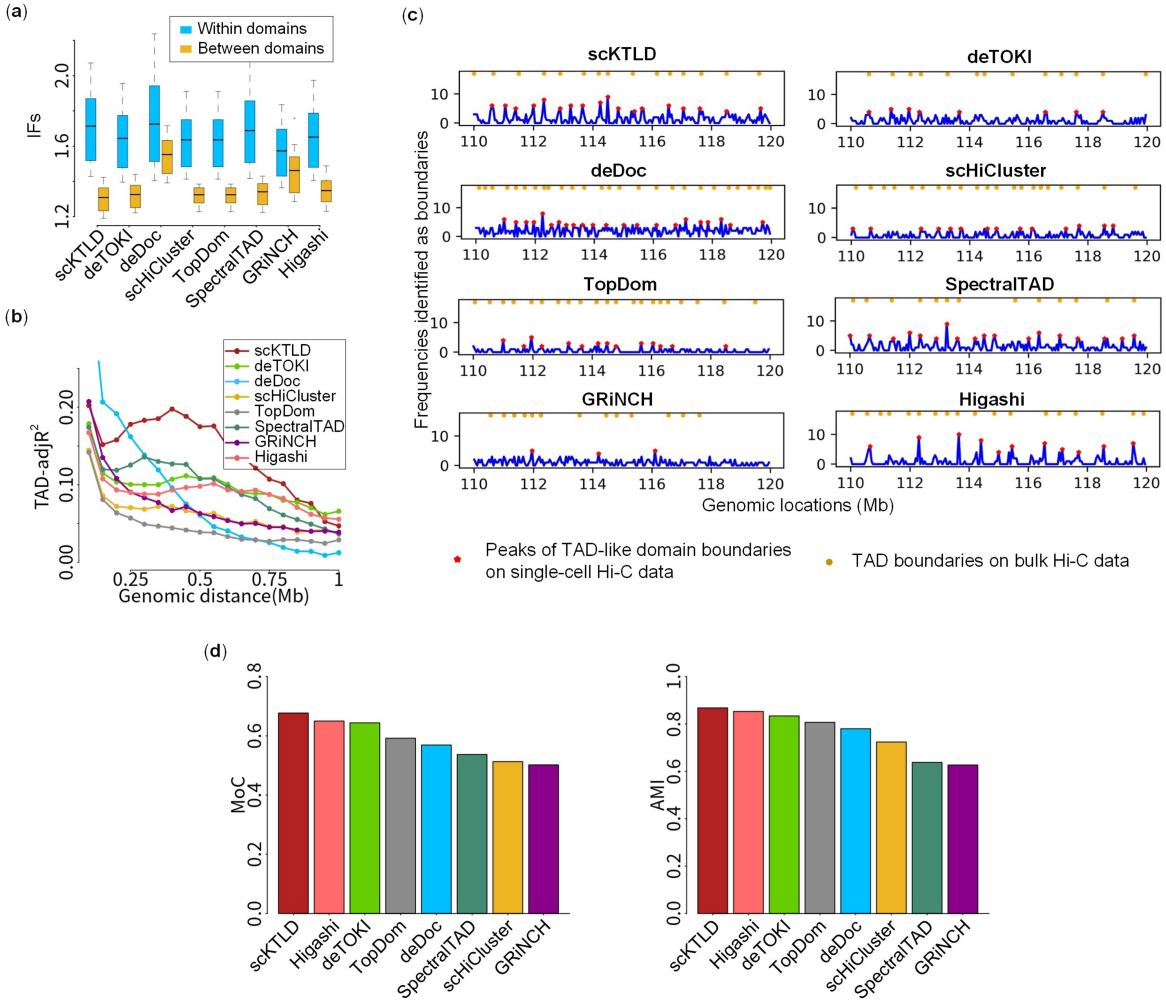
Results on experimental single-cell Hi-C data. The TAD-like domains were called by scKTLD and the other seven methods, including deTOKI, deDoc, scHiCluster, TopDom, SpectralTAD, GRiNCH, and Higashi, on single-cell Hi-C contact matrices for chromosome 1 of 17 GM12878 single cells from Tan’s dataset at 50 kb resolution. (a) Distribution of the IFs within TAD-like domains versus that between adjacent TAD-like domains. (b) Accuracy of domain assembly. The variation in the IFs stratified by genomic distance can be explained by the classification of TAD-like domains; the proportion of this variation within 1 MB of genomic distance is calculated (measured by TAD-adjR^2^) to quantify the accuracy of TAD assembly. In addition, the corresponding bulk Hi-C contact matrices for chromosome 1 of the GM12878 cell line (GSM1551550_HIC001) from Rao’s dataset at 50 kb resolution were also involved to investigate the relationship between TAD-like domains on single-cell Hi-C data and TADs on bulk Hi-C data. (c) Profile of the frequencies at which the bins are identified as the boundaries of TAD-like domains on single-cell Hi-C data (110–120 Mb); the peaks on this profile and the boundaries of TADs called on the corresponding bulk Hi-C data are marked. (d) Similarity between the bins as the peaks of TAD-like domain boundaries on single-cell Hi-C data and the TAD boundaries called on the corresponding bulk Hi-C data; the similarity is scored using MoC and AMI.

Beyond the downsampled bulk Hi-C data and simulated single-cell Hi-C data, TAD-like domains were also called on single-cell Hi-C data from Tan’s and Flyamer’s experiments. Unlike the simulated single-cell Hi-C data, the ground-truth TAD-like domains on experimental data are unknown, and there is no gold standard to score the accuracy of identified TAD-like domains. Thus, we attempted to assess the quality of TAD-like domains in the following ways:. One is the distribution of the IFs within TAD-like domains versus that between adjacent TAD-like domains. As shown in Fig. 4a and Supplementary Fig. S6, the IFs within TAD-like domains are higher than those between adjacent TAD-like domains for all the methods except for GRiNCH on two datasets, ZygMs and ZygPs, and our scKTLD has the highest value within TAD-like domains as well as the lowest value between adjacent TAD-like domains in most cases, indicating that the block structure of TAD-like domains called by scKTLD seems more clear. Another is the accuracy of domain assembly. If TAD-like domains are accurately called, one would expect that a high proportion of the variation in the IFs can be explained by the classification of TAD-like domains, and a metric named TAD-adjR^2^ has been used to quantify this proportion to investigate the accuracy of domain assembly (An *et al.* 2019). Given this, the TAD-adjR^2^ for different methods within 1 Mb genomic distance is shown (Fig. 4b and Supplementary Fig. S7). Generally, our scKTLD has a higher TAD-adjR^2^, especially in the range from 0.25 to 0.75  MB. The third is the relationship between TAD-like domains on single-cell Hi-C data and TADs on bulk Hi-C data. The frequencies at which the bins are identified as the boundaries of TAD-like domains across different single cells are counted and compared with TAD boundaries called on bulk Hi-C data. It can be seen that the frequency of boundaries is greater than zero at most genomic bins for single cells, which is in line with the cell-to-cell variation of TAD-like domains. Interestingly, these boundaries of the TAD-like domain are not completely random; it seems that the TAD-like domain boundaries on single-cell Hi-C data prefer to be located at the bins, which are also the TAD boundaries on bulk Hi-C data (Fig. 4c). This observation is in agreement with the results obtained by an imaging technology for chromatin organization tracing with kilo-base and nanometer-scale resolution (Bintu *et al.* 2018). Furthermore, to score the reproducibility between the boundaries called on single-cell and bulk Hi-C data, the bins as the peaks of TAD-like domain boundaries are regarded as the consensus boundaries for single cells, and the similarity between these consensus boundaries and TAD boundaries called on bulk Hi-C data was scored using MoC and AMI. It is shown that scKTLD and Higashi can reach both the higher MoC and AMI (Fig. 4d), indicating that the boundaries given by them have better reproducibility across single-cell and bulk Hi-C data.

### 3.4 Conservation of TAD-like domain boundaries between cells

**Figure 5.**
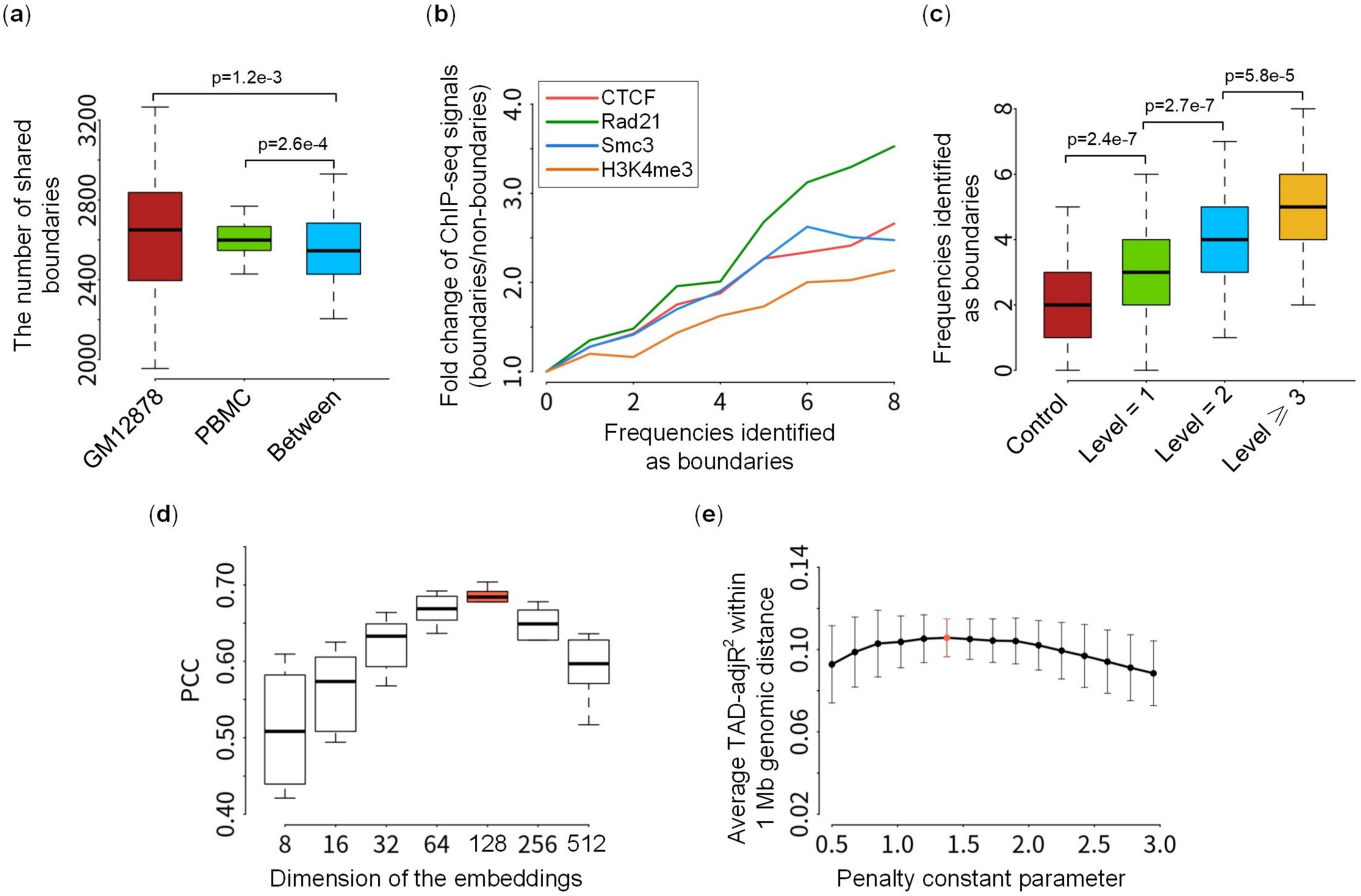
Biological relevance of TAD-like domains called by scKTLD and tuning of two main hyperparameters. (a) Boxplot of the number of shared TAD-like domain boundaries within each of the two cell types and between them. The *P* values were calculated via a one-sided Wilcox test. (b) Curve of the enrichment of architectural proteins and histone marks at TAD-like domain boundaries versus the frequencies at which the bins are identified as boundaries; the enrichment is scored by a fold change of the average ChIP-seq signals at TAD-like domain boundaries to that at the bins not identified as boundaries in any cell. (c) Boxplot of the frequencies at which the bins are identified as TAD-like domain boundaries on single-cell Hi-C data; these frequencies are divided into three groups using the hierarchical levels of corresponding bins on bulk Hi-C data; control indicates the bins not identified as TAD boundaries on bulk Hi-C data; and the *P* values were also calculated via a one-sided Wilcox test. In addition, based on the full and downsampled bulk Hi-C data of the GM12878 cell line from Rao’s experiment, the tuning of two main hyperparameters was examined. (d) Boxplot of the Pearson correlation coefficient between the original full bulk Hi-C contact matrix and the corresponding artificial contact matrix reconstructed by the embeddings with different dimensions; the embeddings were obtained from the downsampled Hi-C contact matrix. (e) Curve of the average TAD-adjR^2^ (mean ± SD) within 1 Mb genomic distance versus the penalty constant parameter in changepoint detection. The average TAD-adjR^2^ is calculated on a downsampled Hi-C contact matrix. All the domains were called on contact matrices at 50 kb resolution.

It has been reported that TAD boundaries on bulk Hi-C data are conserved across replicates, even across cell lines (Dixon *et al.,* 2012; Durand *et al.,* 2016). Different from the above, single-cell Hi-C data shows cell-to-cell heterogeneities, but the conservation of TAD-like domains for different single cells is not very clear. Considering the ability of scKTLD to handle ultra-sparse single-cell Hi-C contact matrix and identify TAD-like domains on it, our proposed method is used to examine the number of TAD-like domain boundaries shared between single cells from Tan’s experiment, including 17 GM12878 cells and 18 PBMC cells. As shown in Fig. 5a, a considerable number of TAD-like domain boundaries are shared between cells within and across cell types, and the number of shared boundaries within GM12878 and PBMC cells seems greater than that between them, which can be further quantified by *P* values (1.2e−3 < 0.05 for GM12878 and 2.6e−4 < 0.05 for PBMC). It shows that the cells within the same cell type share more consensus TAD-like domain boundaries than those across types. To gain a deeper understanding of the conservation and heterogeneity of TAD-like domains in single cells within and across cell types, taking a specific genomic region on chromosome 1 as an example, the heatmaps of merged single-cell Hi-C contact matrices and the corresponding profiles of insulation score (Crane *et al.* 2015), as well as the positions of TAD-like domain boundaries called by scKTLD for each single cell, were shown (Supplementary Fig. S8). It can be found that the TAD-like domain boundaries are heterogeneous in single cells, regardless of cell types. Interestingly, this heterogeneity appears to vary across different genomic locations. For the regions marked with blue boxes, the boundaries seem to exhibit vibrations around certain assumed anchor points, and the vibrations do not show obvious differences between cells across different types, indicating that these domain boundaries are conserved across cell types. In contrast, for the regions marked with orange boxes, the boundaries that appear in GM12878 cells seem to shift or disappear in PBMC cells, which can also be validated by the profile of the insulation score. This suggests that these domain boundaries exhibit more conservation within cell types. Overall, the observations may explain the conservation and heterogeneity of TAD-like domain boundaries in single cells. Despite the high heterogeneity of TAD-like domain boundaries among cells, they maintain the stability of their basic domain architectures while performing their own functions.

### 3.5 Biological relevance of TAD-like domains

To investigate the biological relevance of the TAD-like domains called by scKTLD on single-cell Hi-C data, one cell was randomly chosen from Tan’s GM12878 dataset, and the average ChIP-seq signals, including CTCF, Rad21, Smc3, and H3K4me3, per bin within 500 kb up-stream and down-stream flanking regions of each TAD-like domain boundary were calculated (Supplementary Fig. S9). As expected, the boundaries are enriched by these architectural proteins and histone marks, which is consistent with the results given by the imaging technology for tracing chromatin organizations at the single-cell level, where the TAD-like domain boundaries are more likely to appear at CTCF and Rad21 peaks (Bintu *et al.* 2018, Zhou *et al.* 2019). Herein, the enrichment is scored by a fold change of the average ChIP-seq signals at the boundaries to that at the bins not identified as boundaries in any cell, and the relationship between the enrichment and the frequencies at which the bins are identified as boundaries across different single cells was examined (Fig. 5b). Generally, this fold change is related to the frequency; the higher the frequency, the greater the fold change. That is to say, the bins identified as TAD-like domain boundaries with higher frequency may be more enriched for these architectural proteins and chromatin marks. This interesting observation encourages us to further explore the biological meaning underlying the enrichment. In light of some reports that higher enrichment of architectural proteins and histone marks is usually accompanied by higher levels of hierarchical TADs on bulk Hi-C data (Wang *et al.* 2017, An *et al.* 2019, Cresswell *et al.* 2020, Liu *et al.* 2022), we speculate that there might be an association between TAD-like domains on single-cell Hi-C data and hierarchical TADs on bulk Hi-C data. To check this point, the hierarchical TADs were called with a recently developed tool named TADfit (Liu *et al.,* 2022), which is a multivariate regression model for the identification of hierarchical TADs on replicate Hi-C data. And the hierarchical level of a bin can be assigned using the terminology given by An *et al.* (2019), where the boundary level of a bin is defined as the maximum number of hierarchical TADs that use a boundary on either its left or right side. Then, for the bins identified as TAD boundaries with different hierarchical levels on bulk Hi-C data, the frequencies at which these bins are identified as TAD-like domain boundaries across different single cells were also counted. As shown in Fig. 5c, the TAD boundaries on bulk Hi-C data are more frequently identified as TAD-like domain boundaries on single-cell Hi-C data, and this trend seems more obvious for the TAD boundaries with higher hierarchical levels. In other words, TAD-like domain boundaries in single cells preferentially occur at TAD boundaries in bulk cells, especially at those with higher hierarchical levels.

### 3.6 Runtime and memory consumption

The efficiency of TAD-like domain identification is also important, since there are often a large number of individual cells produced in a single-cell Hi-C experiment. Thus, the runtime and memory consumption of scKTLD are compared with those of the other seven methods on Tan’s and Flyamer’s single-cell Hi-C datasets on a computing platform of Ubuntu 18.04 LTS with an Intel (R) Xeon (R) E5-2609 @ 1.70 GHz CPU and an NVIDIA GeForce RTX 3090 GPU. scHiCluster and deTOKI were executed on multiple threads of 4, 8, and 12 since they are designed to support multi-threaded parallel computing. As shown in Supplementary Table S5, it can be seen that scKTLD runs faster and consumes less memory than most of the methods except for TopDom and SpectralTAD. As for the multi-threaded scHiCluster and deTOKI, as the number of threads increases, the two methods sacrifice more memory to speed up the runtime, showing an obvious acceleration effect with multi-threading. However, even when the number of threads is increased to 12 (limited by our computing platform), they still run much slower than single-threaded scKTLD. Therefore, scKTLD is competitive in runtime and memory consumption and outperforms deTOKI, scHiCluster, and Higashi, which are designed for single-cell Hi-C data. This competitiveness may be explained by the following three points: (i) randomized tSVD (Halko *et al.* 2011) to avoid singular value decomposition of the large proximity matrix in the initialization of graph embedding. (ii) Chebyshev expansion to avoid the eigen-decomposition of the large Laplacian matrix during the spectral propagation. And (iii) PELT to find the optimal changepoints within a linear time consumption.

## 4 Discussion

There are two main hyperparameters that need to be tuned in SCKTLD. One is the dimension of the embeddings, and the other is the penalty constant *β* in changepoint detection. In this paper, the dimension is chosen according to the correlation between the original full-bulk Hi-C contact matrix and the artificial contact matrix reconstructed with the embeddings obtained from the downsampled one. As we can see, the Pearson correlation coefficient (PCC) increases as the dimension goes up from 8 to 128, indicating that more details in genomic structures can be learned in the graph embedding procedure. However, once the dimension exceeds 128, the PCC begins to decrease due to the involvement of more noises (Fig. 5d and Supplementary Fig. S10). Thus, a dimension of 128 was regarded as optimal. In addition, the penalty constant *β* controls the trade-off between segmentation complexity and goodness-of-fit in the changepoint detection procedure. While *β* is set to a smaller value, more TAD-like domains with small sizes will be captured, and while β is set to a larger value, more small domains will trend to be discarded. Herein, the value of *β* is optimized so that the average TAD-adjR^2^ can be maximized across different genomic distances; thus, a value of 1.42 was chosen in this paper (Fig. 5e and Supplementary Fig. S11).

Matrix balancing is widely used for the normalization of bulk Hi-C data, and it has been demonstrated that ICE and KR normalization have an effect on improving the reproducibility between TADs called on bulk Hi-C replicates in our previous benchmark (Lyu *et al.* 2020). So we explored how these normalizations affect the identification of TAD-like domains on ultra-sparse Hi-C data via our scKTLD. To investigate this point, firstly, we compared the similarity of TADs identified by scKTLD on the full and downsampled bulk Hi-C data with the preprocessings of ICE normalization, KR normalization, and no normalization. As shown in Supplementary Fig. S12, the similarity between the TADs identified on the full and downsampled bulk Hi-C data normalized by ICE or KR decreases compared to that without normalization, especially for the AMI metric. Secondly, we also compared the accuracy of domain assembly using the metric TAD-adjR^2^ on Tan’s experimental single-cell Hi-C data with the same preprocessings as above. As shown in Supplementary Fig. S13, it seems that the TAD-adjR^2^ also shows a slight decrease on the normalized single-cell Hi-C data regardless of ICE and KR normalization. These suggest that the results of TAD-like domain identification benefit little from matrix balancing preprocessings for ultra-sparse single-cell Hi-C data. This might be related to the fact that the matrix balancing methods tend to delete ultra-sparse rows and columns, resulting in the deletion of a substantial portion of the contact matrix in the case of single-cell Hi-C. Therefore, they may not work well under the condition of ultra-sparsity (Li *et al.* 2020).

Although we have shown the ability of scKTLD to capture and recover TAD-like domains from ultra-sparse single-cell Hi-C data, it is not clear whether the reconstructed matrix could recover finer structures in single-cells, like long-range interactions. To investigate this, the computational tool HICCUPS (Rao *et al.* 2014) was used to identify the interactions on Hi-C contact matrices, and the interactions between loci that are more than 1.5 Mb apart in genomic coordinates are filtered out as long-range interactions. Thus, the analysis can be carried out in two aspects. Firstly, we called long-range interactions on the full bulk Hi-C contact matrices for chromosome 1 of the GM12878 cell line in Rao’s dataset and made a comparison with those called on the reconstructed Hi-C contact matrices with different embedding dimensions. The comparison was conducted in terms of frequency and intensity, characterized by the number of long-range interactions and fold enrichment score from the donut background, respectively. As shown in Supplementary Fig. S14, it seems that there are much more long-range interactions called on the reconstructed contact matrices, and the fold enrichment scores on them are lower than those on the full bulk Hi-C contact matrices, regardless of the embedding dimensions. It shows that the reconstructed contact matrices still have room for improvement in recovering the long-range interactions to the quality of full bulk Hi-C. Secondly, for the long-range interactions called on the full bulk Hi-C contact matrix, we visualized them by aggregate peak analysis (APA) on the reconstructed ones and made an intuitive comparison with the APA on the full bulk Hi-C. As shown in Supplementary Fig. S15, a significant aggregate enrichment of interactions can be seen on the peak center of the full bulk Hi-C contact matrix, and the enrichment can be weakly observed on the reconstructed ones with higher embedding dimensions. This indicates that our graph embedding procedure has a limited ability to capture long-range interactions. Overall, the embeddings produced by scKTLD may be more suitable for recovering and enhancing the chromatin structures at the TAD level, and the methods that can reliably recover the fine structures, like long-range interactions, are still expected to further reveal the chromatin architectures and their functions in single cells in the future.

## Supplementary Material

btae138_Supplementary_Data

## Data Availability

Our method, scKTLD, has been implemented in a Python package and is available at https://github.com/lhqxinghun/scKTLD.
