# Supplementary material for: Identifying TAD-like domains on single-cell Hi-C data by graph embedding and changepoint detection
Source: Bioinformatics. 2024 Mar 6;40(3):btae138. doi: 10.1093/bioinformatics/btae138 (PMC10960928; doi:10.1093/bioinformatics/btae138)
Supplement: btae138_Supplementary_Data [file btae138_supplementary_data.pdf]

Supplementary Material for

Identifying TAD-like domains on single-cell Hi-C data by graph embedding and changepoint detection

Erhu Liu[1], Hongqiang Lyu[2,*], Yuan Liu[2], Laiyi Fu[2], Xiaoliang Cheng[3], Xiaoran Yin[4]

[1] School of Information and Control Engineering, Xi'an University of Architecture and Technology, Xi'an, China
[2] School of Automation Science and Engineering, Faculty of Electronic and Information Engineering, Xi'an Jiaotong University, Xi'an, China
[3] Department of Pharmacy, The First Affiliated Hospital of Xi'an Jiaotong University, Xi'an, China
[4] Department of Oncology, The Second Affiliated Hospital of Xi'an Jiaotong University, Xi'an, China
*Corresponding author. Email: hongqianglv@mail.xjtu.edu.cn

I. Supplementary Methods

II. Supplementary Figures

III. Supplementary Tables

IV. References

I. Supplementary Methods

Simulation of single-cell and reference Hi-C data

We used the following procedures to simulate single-cell and reference Hi-C contact matrices from the three-dimensional physical models of chromosomes. First, we selected two distant fragments (25 Mb – 30 Mb and 100 Mb – 105 Mb) on chromosome 8 of K562 cell line at random in Rao's bulk Hi-C dataset to mimic two types of cells, respectively, and 100 three-dimensional physical models were generated from each segmented contact matrix of bulk Hi-C with the help of Integrative Modeling Platform (Bau and Marti-Renom, 2012; Serra, et al., 2017). Then for each model, the Euclidean distances $Dist_{i,j}$ between every loci pair (i, j) are calculated, and four contact matrices are generated by weighted sampling the genomic loci, including three single-cell contact matrices corresponding to three minimal contacting distances (500, 750 and 1000) and one reference contact matrix to define the ground-truth TADs (Supplementary Table S2).

For single-cell Hi-C contact matrix, the weight between loci pair (i, j) is $W_{i,j} = D_{th} - Dist_{i,j}$ with the D_{th} representing the distance threshold, and only the loci within D_{th} (or $W_{i,j} > 0$) is considered to contact. The expected number of contacts between loci pair (i, j) is regarded proportion to $W_{i,j}$ and can be calculated by setting the total number of contacts to 1000 per 5 Mb genomic region, close to the experimental single-cell Hi-C data (Tan, et al., 2018). Then the contacts between every loci pair can be simulated by randomly sampling from Binomial distributions with the expected number of contacts without replacement. For reference Hi-C contact matrix, the weight between loci (i, j) is $W_{i,j} = \frac{1}{Dist_{i,j}}$ and the expected number of contacts between them is regarded proportion to $W_{i,j}$, which can be calculated given that the total number of contacts is about 0.35M per 5 Mb genomic region, close to the experimental bulk Hi-C (Rao, et al., 2014). Similarly, the contacts between every loci pair can be simulated by randomly sampling from Poisson distributions with the expected number of contacts without replacement. To get Hi-C contact matrices at resolution of 50 kb, the simulated Hi-C contact matrices were further binned into 50 kb for TAD-like domain calling.

Chebyshev expansion for the modulated Laplacian

To avoid the expensive computational cost in explicit eigen-decomposition of the Laplacian matrix for large graphs, we used truncated Chebyshev expansion to speed up the calculation of the modulated Laplacian, which has been proven to have the ability to approximate the modulated Laplacian well in a fast way (Kipf and Welling, 2016; Zhang, et al., 2019). For Chebyshev polynomials of the first kind, we have $T_0(x) = 1$, $T_1(x) = x$ and the higher order can be obtained by iterating the formula $T_{i+1}(x) = 2xT_i(x) - T_{i-1}(x)$. Herein we define a polynomial transformation $\bar{\lambda} = \frac{1}{2}\left[(\lambda - \mu)^2 - 1\right]$, then the modulator function will be $g(\lambda) = f(\bar{\lambda}) = e^{-\bar{\lambda}\theta}$ and the modulated Laplacian can be approximated by Chebyshev polynomials with the following forms:

$$\mathbf{L} \approx \mathbf{U}\sum_{i=0}^{k-1} c_i(\theta) T_i(\bar{\mathbf{\Lambda}})\mathbf{U}^{-1} = \sum_{i=0}^{k-1} c_i(\theta) T_i(\bar{\mathbf{L}}) \quad (1)$$

where $\bar{\mathbf{\Lambda}} = \frac{1}{2}\left[(\mathbf{\Lambda} - \mu\mathbf{I}_n)^2 - \mathbf{I}_n\right]$, $\bar{\mathbf{L}} = \frac{1}{2}\left[(\mathbf{L} - \mu\mathbf{I}_n)^2 - \mathbf{I}_n\right]$ and $c_i(\theta)$ is the coefficient of the Chebyshev expansion for $f(\bar{\lambda})$, which can be easily calculated according to the orthogonality of $T_i(x)$ with the weight $1/\sqrt{1-x^2}$ on the

interval $[-1, 1]$. k is the truncated order, which is set to 10 in this study. After obtaining these coefficients, $\mathbf{L}$ can be approximated as:

$$\mathbf{L} \approx B_0(\theta)T_0(\bar{\mathbf{L}}) + 2\sum_{i=1}^{k-1}(-1)^i B_i(\theta)T_i(\bar{\mathbf{L}}) \tag{2}$$

where $B_i(\theta)$ is the modified Bessel function of the first kind. The equation (2) enables us to calculate $\mathbf{L}$ and propagate the embeddings in the spectrally modulated network very efficiently. Finally, to maintain the orthogonality of the original embedding space, SVD is performed again on the propagated embeddings.

PELT optimization

PELT considers each observation $\mathbf{y}_t$ sequentially and use an explicit pruning rule to determine whether or not to discard it from the set of potential changepoints (Killick, et al., 2012). Let $c\left(\mathbf{y}_{(\tau_{i-1}+1)..\tau_i}\right) = \sum_{t=\tau_{i-1}+1}^{\tau_i} \left\| \phi(\mathbf{y}_t) - \bar{\boldsymbol{\mu}}_{(\tau_{i-1}+1)..\tau_i} \right\|_{\mathcal{H}}^2$ be the measure of fit for the observation from $\tau_{i-1}+1$ to τ_i. Then PELT can be specified as follows in Supplementary Algorithm 1.

Supplementary Algorithm 1 PELT

Input: sequential vectors $\{\mathbf{y}_t\}_{t=1}^n$, a measure of fit $c(\cdot)$, penalty constant β.

Initialize $Z[0] = -\beta$, $L[0] = \varnothing$ and $\chi = \{0\}$.

for $t = 1,\ldots,n$ **do**

  $\hat{t} = \arg\min_{s\in\chi} \{Z[s] + c(\mathbf{y}_{s..t}) + \beta\}$ // Find the optimal from the candidate changepoints up to index t

  $Z[t] = Z[\hat{t}] + c(\mathbf{y}_{\hat{t}..t}) + \beta$ // Calculate the $Z[t]$

  $L[t] = L[\hat{t}] \cup \{\hat{t}\}$ // Add $\hat{t}$ to changepoint list

  $\chi = \{s \in \chi \mid Z[s] + c(\mathbf{y}_{s..t}) \le Z[t]\} \cup \{t\}$ // Discard the impossible changepoints from set for searching

end for

Output: set $L[n]$ of estimated changepoint indexes.

Configurations of scKTLD and competing methods

scKTLD. Our scKTLD treats symmetric single-cell Hi-C contact matrix as an adjacency matrix for a weighted graph, embeds the graph into a low-dimensional space with the help of sparse matrix factorization followed by spectral propagation, and identifies the TAD-like domains in the embedding space using a kernel-based changepoint detection. To implement this method, the source code can be downloaded from https://github.com/lhqxinghun/scKTLD, and there are two parameters need to be tuned. During the implementation of TADfit in this study, we set the embedding dimension to 128 and the penalty constant to 1.42 by default.

deTOKI. deTOKI uses a sliding window to segment the entire chromosome into smaller sub-matrices spanning 8 Mb genomic regions. Then a consensus map is constructed by multiple non-negative matrix factorizations (NMF), and the TAD-like domain boundaries can be determined based on the cluster rate (CR) calculated on the consensus map. To implement this method, the program TOKI was downloaded from https://github.com/lixiaoms/TOKI, and two main parameters need to be specified. We set the resolution to the bin size of input contact matrices, and set the TAD mean size (measured in kb) to (600, 800) by default. Besides, deTOKI supports multi-threaded execution, and we set the

number of threads to 4, 8, and 12 to evaluate its computational efficiency with multiple threads.

deDoc. deDoc is a TAD detection algorithm based on the theories of structural information and graph. It seeks to extract a structure that minimizes the global uncertainty of the Hi-C graph. To implement this method, the program deDoc was download from https://github.com/yinxc/structural-information-minimisation, and no parameters need to be specified to run it. deDoc integrates two algorithms, denoted as deDoc(E) and deDoc(M), where deDoc(M) reapplies deDoc(E) to its identified modules iteratively according to the proposer. In our test, deDoc(M) typically produce extremely small TADs on single-cell Hi-C data, occupying only about 2 bins. Therefore, we used deDoc(E) for comparison.

TopDom. TopDom is a TAD detection algorithm based on statistics. It calculates a binSignal for each bin by averaging the interaction frequencies upstream and downstream, and determines the TAD boundaries by local minima calling and statistic filtering. In our comparison, TopDom was performed using the R package TopDom (0.10.1), which can be downloaded from CRAN repository, and two parameters need to be specified to execute it. window.size was set to 10 and statFilter was set to true according to the instructions for the package.

scHiCluster. scHiCluster uses convolution and random walk with restart to impute the sparse single-cell Hi-C contact matrix, and only top-ranked interactions preserved after imputation. Then it employs TopDom to call the TAD-like domains on the imputed contact matrices. To implement this method, the python package scHiCluster was downloaded from https://github.com/zhoujt1994/scHiCluster, and there are five parameters need to be tuned. We configured these parameters by default as follows: pad=1, rp=0.5, prct=20, window.size =10, and statFilter=true.

SpectralTAD. SpectralTAD identifies hierarchical TADs by a modified version of the multiclass spectral clustering algorithm. The initial TADs are obtained by maximizing an average silhouette score, and the hierarchical structures are then determined by iteratively portioning the initial TADs. This method was implemented using R package SpectralTAD (v. 1.2.0) which can be downloaded from BiocManager. During the implementation, the levels of hierarchical structures was set to 1 since we only consider basic TAD-like domains in single cells in our study. The parameter qual_filter was set to false, and min_size was set to 5 by default.

GRiNCH. GRiNCH employs graph regularized NMF to obtain the lower-dimension representation of a high-dimensional Hi-C contact matrix while capturing the distance dependence of interaction frequencies, and chain-constrained k-medoids clustering is performed to find TADs. The program of GRiNCH was downloaded from https://roy-lab.github.io/grinch/, and there are four parameters need to be tuned: the number of clusters, expected size of a cluster, neighborhood radius, and regularization strength. During the implementation of GRiNCH, we set these parameters to the default values recommended by the software.

Higashi. Higashi transforms the input single-cell Hi-C data into a hypergraph, where each hyperedge connects one cell node and two bin nodes, and imputes the single-cell Hi-C contact matrices by predicting missing hyperedges within the hypergraph. The TAD-like domains are then called by finding the local minima of the insulation score on the imputed contact matrices. To implement Higashi, the source code was downloaded from https://github.com/ma-compbio/Higashi, and there are 14 parameters required to be tuned. According to the configure file given by the original proposer, we set these parameters as follows: resolution=50 kb or 25 kb (according to the bin size), minimum_distance = 2000000, maximum_distance = -1, local_transfer_range = 1, dimensions = 64, minimum_impute_distance = 0, maximum_impute_distance = -1, neighbor_num = 5, optional_smooth = false, optional_quantile = false, loss_mode = zinb, random_walk = false, window_ins = 500000, window_tad = 500000.

II. Supplementary Figures

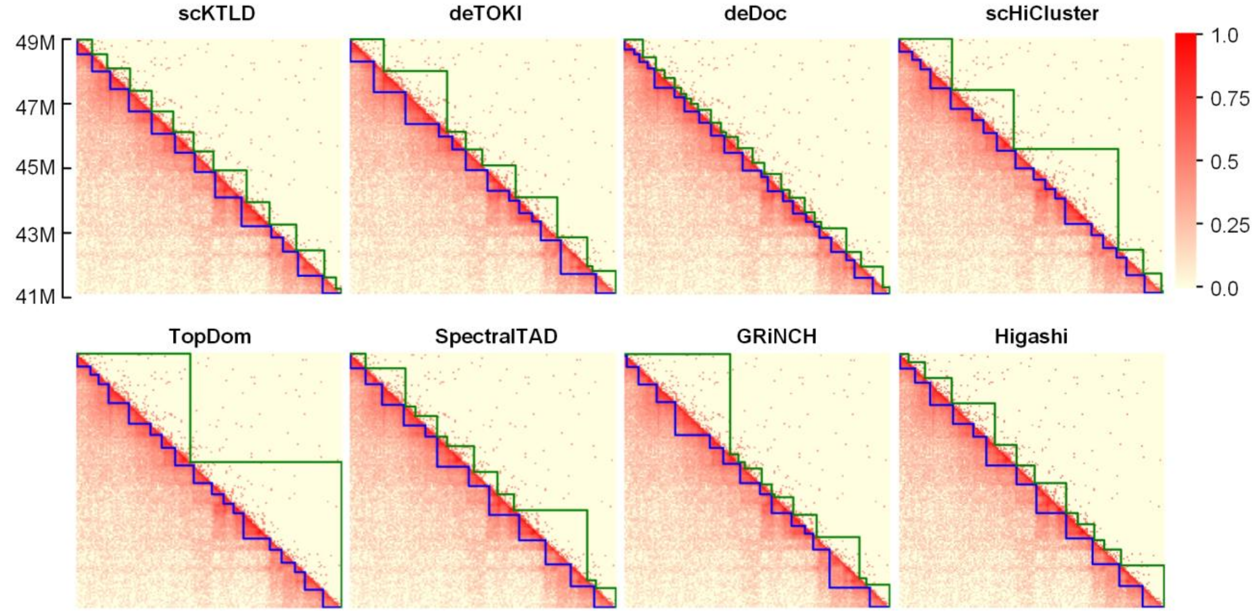

Supplementary Fig. S1. The comparison of TADs called on the full and downsampled bulk Hi-C data. The heatmaps of an artificial contact matrix for chromosome 1 of GM12878 cell line in Rao's dataset (GSM1551550_HIC001, 41 M – 49M,) at 50 kb resolution were shown, with lower left triangle for the full bulk Hi-C and the upper right triangle for the downsampled bulk Hi-C. The identified TADs were marked with blue and green lines on the heatmaps of full and downsampled bulk Hi-C, respectively.

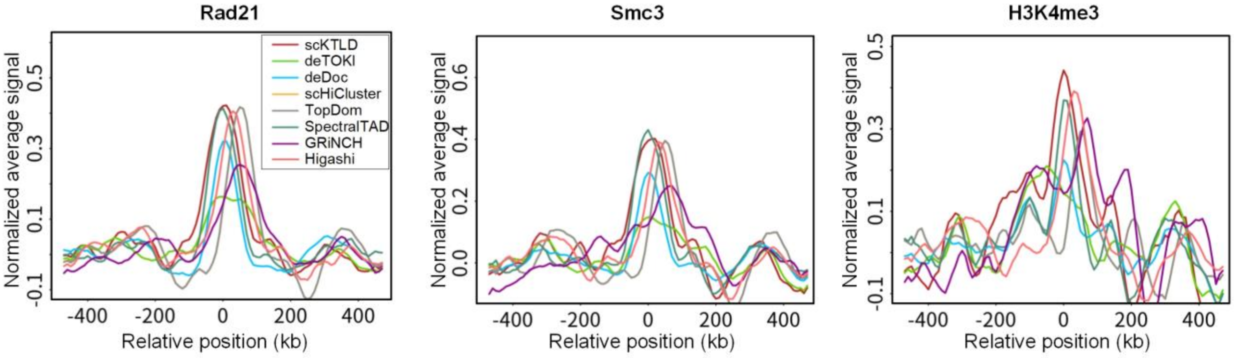

Supplementary Fig. S2. The enrichment of architectural proteins and histone marks at the boundaries called on full bulk Hi-C. The normalized average ChIP-seq signals, including Rad21, Smc3 and H3K4me3, per bin within 500 kb up-stream and down-stream flanking regions of each TAD boundary called on full bulk Hi-C data for chromosome 1 of GM12878 cell line in Rao's dataset at 50 kb resolution were shown.

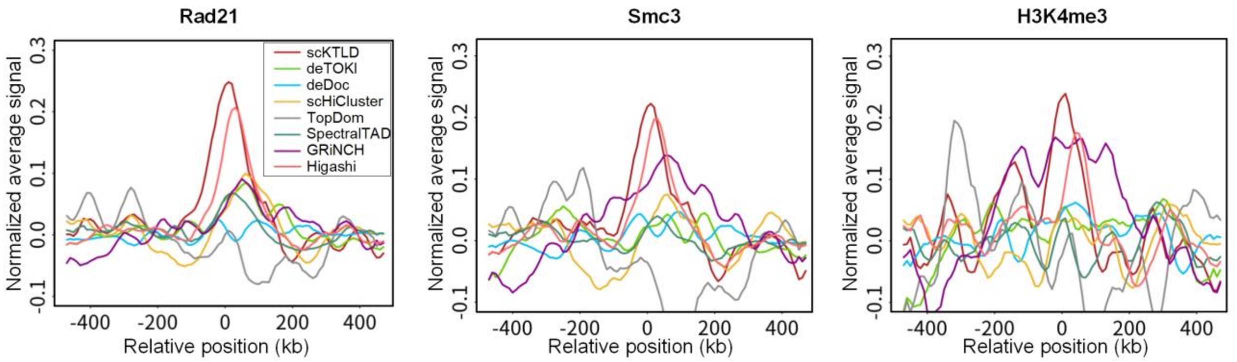

Supplementary Fig. S3. The enrichment of architectural proteins and histone marks at the boundaries called on downsampled bulk Hi-C. The normalized average ChIP-seq signals, including Rad21, Smc3 and H3K4me3, per bin within 500 kb up-stream and down-stream flanking regions of each TAD boundary called on downsampled bulk Hi-C data for chromosome 1 of GM12878 cell line in Rao's dataset at 50 kb resolution were shown.

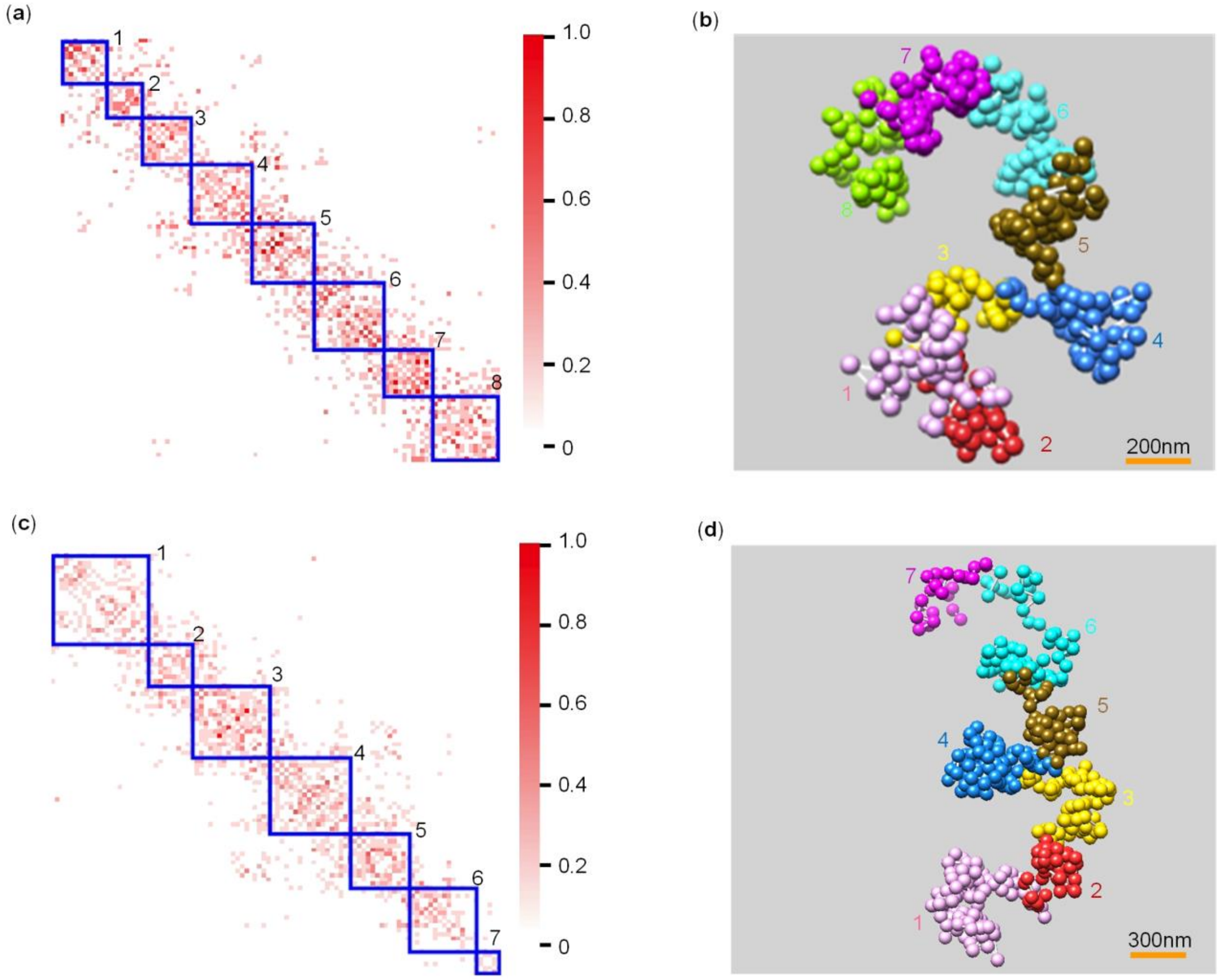

Supplementary Fig. S4. The TAD-like domains visualized on simulated single-cell Hi-C contact matrix and chromosome models. (a), (b) Heatmap of single-cell Hi-C contact matrix and the chromosome model for the 1st type of simulated cell. (c), (d) Heatmap of single-cell Hi-C contact matrix and the chromosome model for the 2nd type of simulated cell. The TAD-like domains are marked with blue squares on heatmaps and are distinguished by different colored spheres on the chromosome models. The contact matrices shown are at 50 kb resolution, and one particle in the chromosome models represents a genomic loci spanning 10 kb.

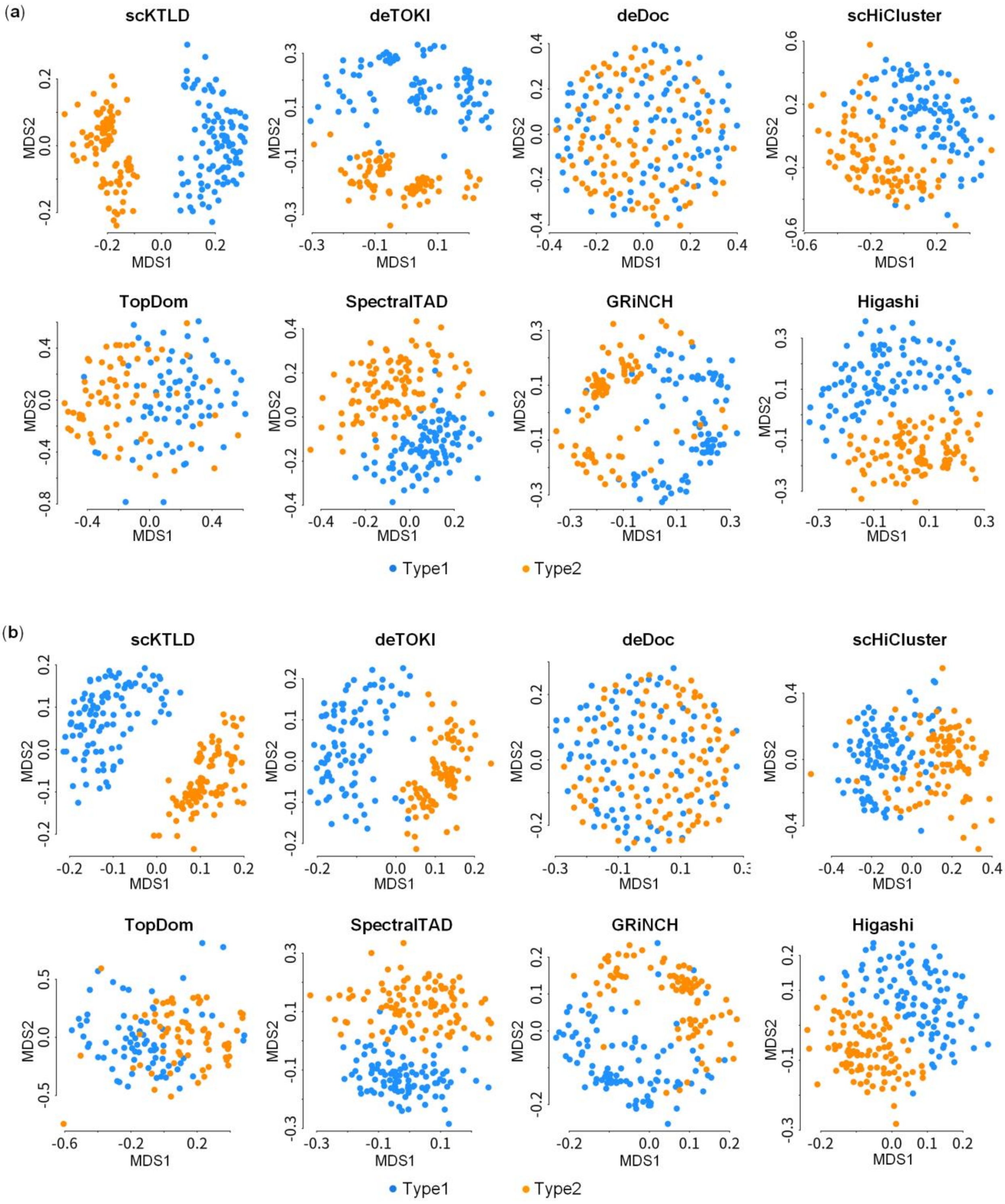

Supplementary Fig. S5. Comparison of scatter plot of simulated single cells in the embedding space. The TAD-like domains were called on the two types of simulated single-cell Hi-C contact matrices at 50 kb resolution and 750 distance threshold, and the cells were then embedded by MDS, where the MoC and AMI between the TAD-like domains called on these cells were employed to score the similarity between cells. (a) Scatter plot of simulated single cells in the embedding space with MoC employed for similarity metric. (b) Scatter plot of simulated single cells in the embedding space with AMI employed for similarity metric.

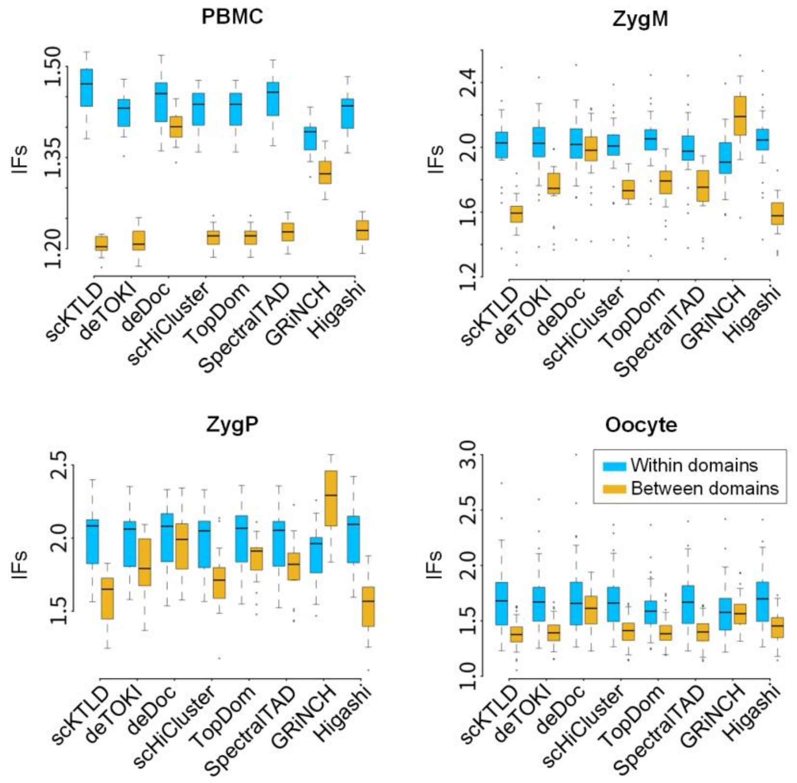

Supplementary Fig. S6. Distribution of the IFs within TAD-like domains versus that between adjacent TAD-like domains. The TAD-like domains were called on single-cell Hi-C contact matrices for chromosome 1 of 18 PBMC cells from Tan's dataset as well as 20 ZygM, 18 ZygP and 70 Oocyte cells from Flyamer's dataset at 50 kb resolution.

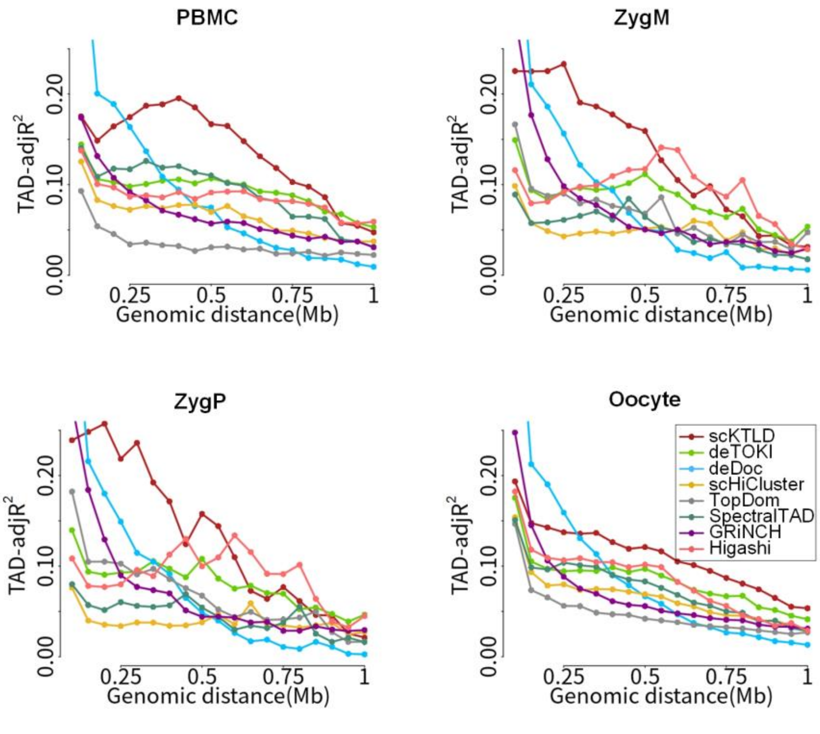

Supplementary Fig. S7. Accuracy of domain assembly. The TAD-like domains were called on single-cell Hi-C contact matrices for chromosome 1 of 18 PBMC cells from Tan's dataset as well as 20 ZygM, 18 ZygP and 70 Oocyte cells from Flyamer's dataset at 50 kb resolution.

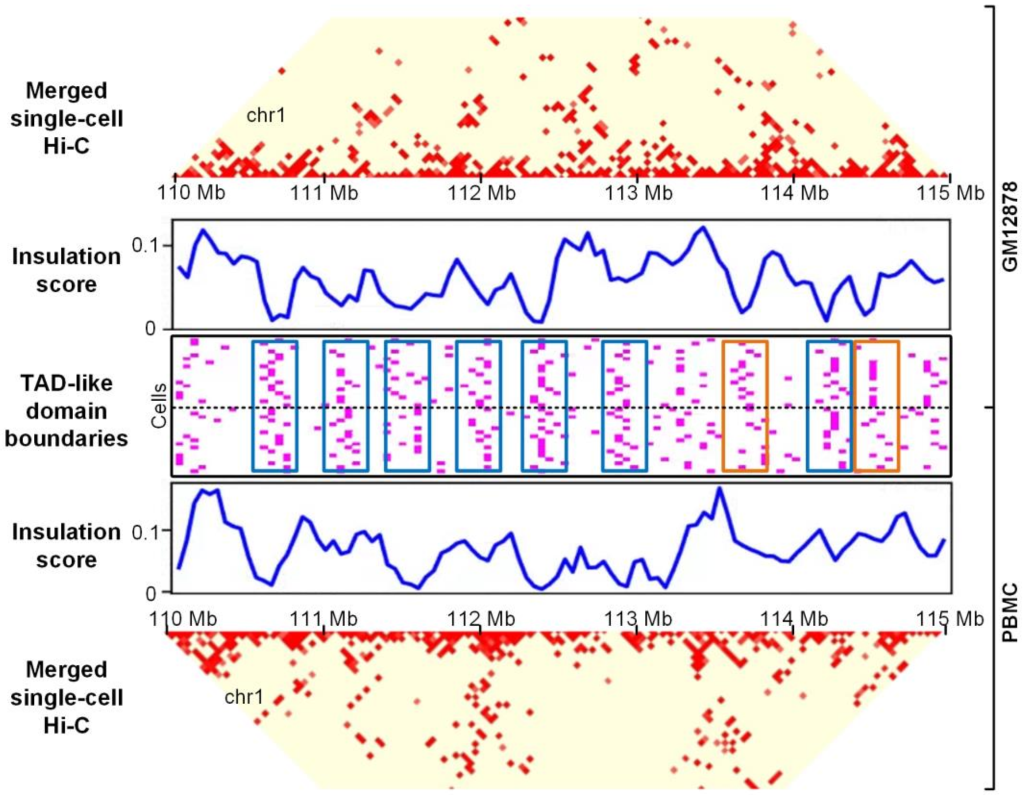

Supplementary Fig. S8. The conservation and heterogeneity of TAD-like domains revealed by scKTLD in single cells. The heatmaps of merged single-cell Hi-C contact matrices for chromosome1 of 17 GM12878 cells and 18 PBMC cells from Tan's dataset at 50 kb resolution (110 Mb – 115 Mb), and the corresponding profiles of their insulation score, as well as the positions of TAD-like domain boundaries called by scKTLD were shown.

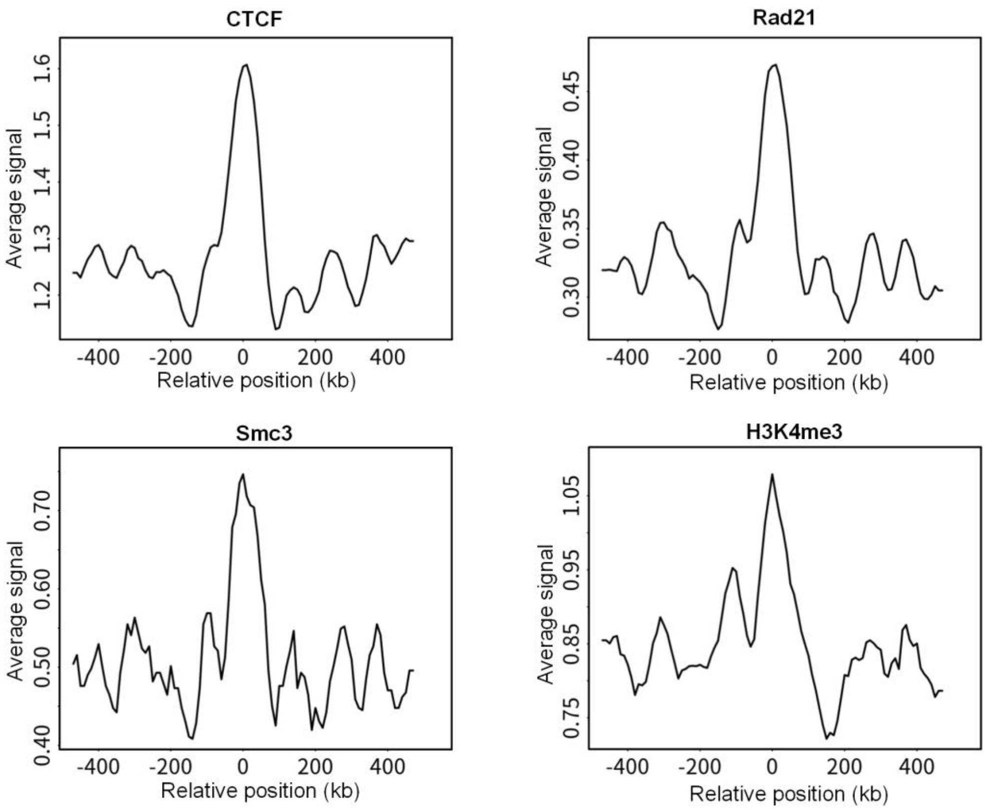

Supplementary Fig. S9. The enrichment of architectural proteins and histone marks at the boundaries identified on single-cell Hi-C data. The average ChIP-seq signals, including CTCF, Rad21, Smc3 and H3K4me3, per bin within 500 kb up-stream and down-stream flanking regions of each TAD boundary called on the single-cell Hi-C contact matrix for chromosome 1 of GM12878 cell #4 in Tan's dataset at 50 kb resolution were shown.

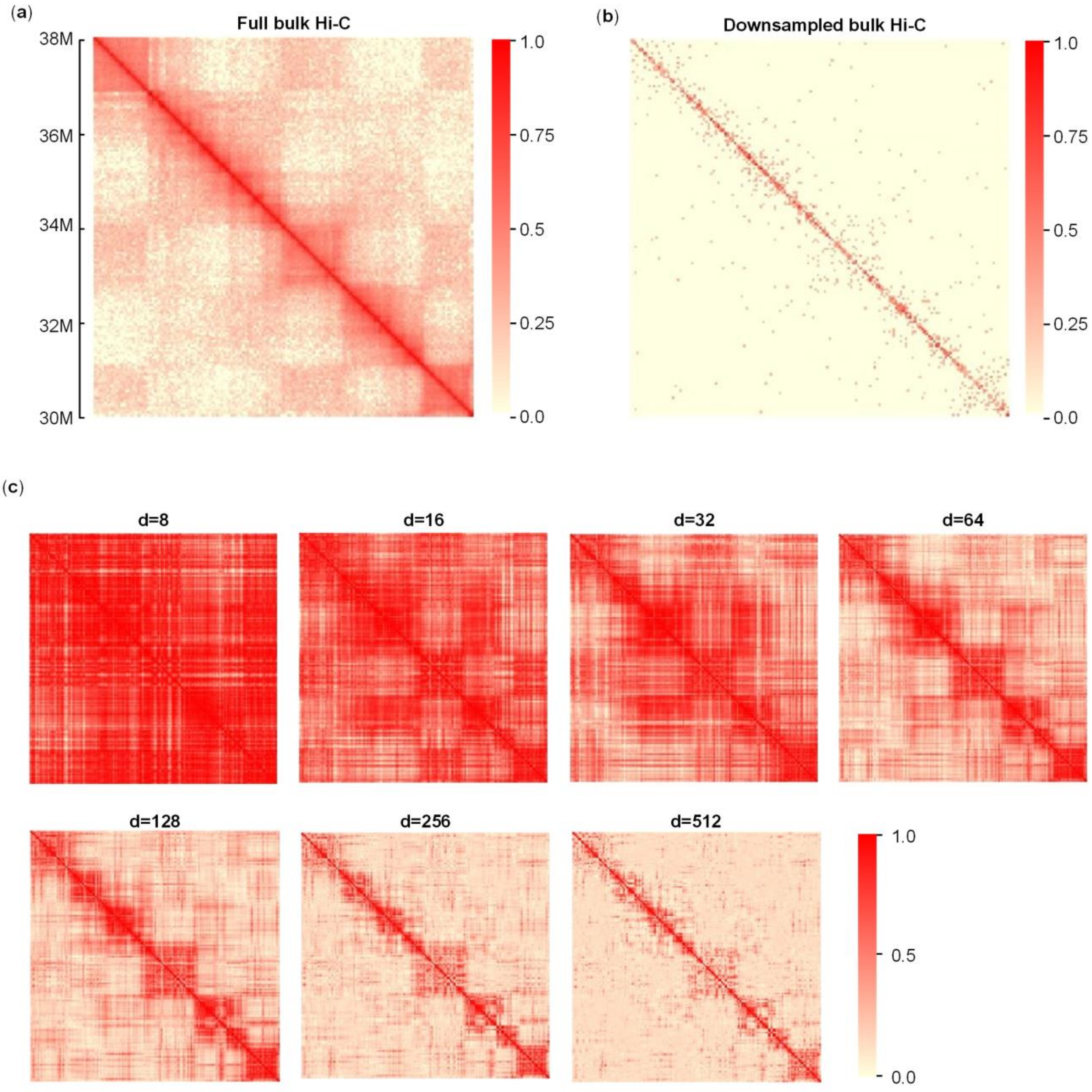

Supplementary Fig. S10. Heatmaps of contact matrix for full bulk Hi-C contact matrix, downsampled bulk Hi-C contact matrix and reconstructed contact matrix. The heatmaps of contact matrices for chromosome 1 of GM12878 cell line (GSM1551550_HIC001, 30M – 38 M) in Rao's dataset at resolution of 50 kb were shown. (a) Heatmap of contact matrix for full bulk Hi-C. (b)Heatmap of contact matrix for downsampled bulk Hi-C. (c)Heatmaps of contact matrices reconstructed with embeddings at different dimensions obtained from the downsampled one.

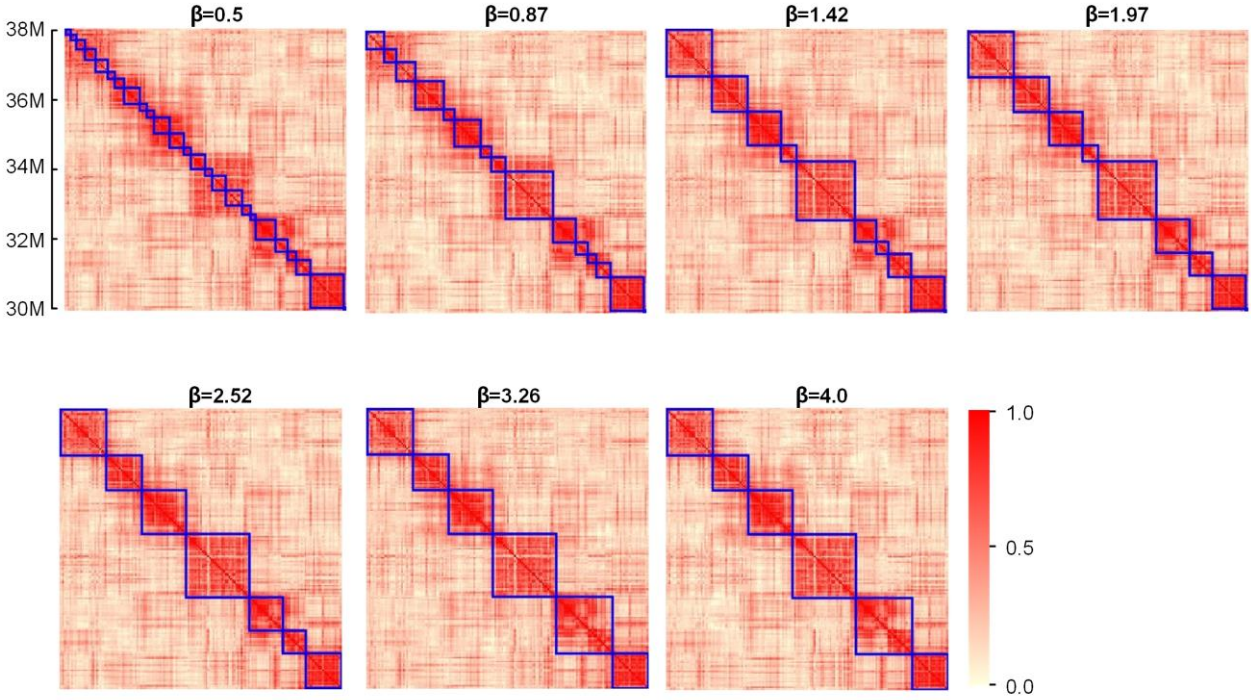

Supplementary Fig. S11. TAD-like domains called with different penalty constants in scKTLD. Heatmaps of contact matrix reconstructed with embeddings obtained from the downsampled bulk Hi-C data of chromosome 1 of GM12878 cell line (GSM1551550_HIC001, 30M – 38M) in Rao's dataset at resolution of 50 kb were shown. The TAD-liked domains called with different penalty constants were shown and marked with blue squares on heatmaps.

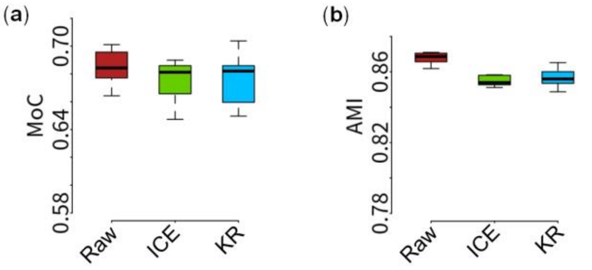

Supplementary Fig. S12. Similarity between the TADs called on the full and downsampled bulk Hi-C data with different normalization preprocessings. (a) Similarity measured by MoC. (b) Similarity measured by AMI. The TADs were called by scKTLD on the Hi-C contact matrices for chromosome 1 of GM12878 cell line from Rao's dataset at 50 kb resolution.

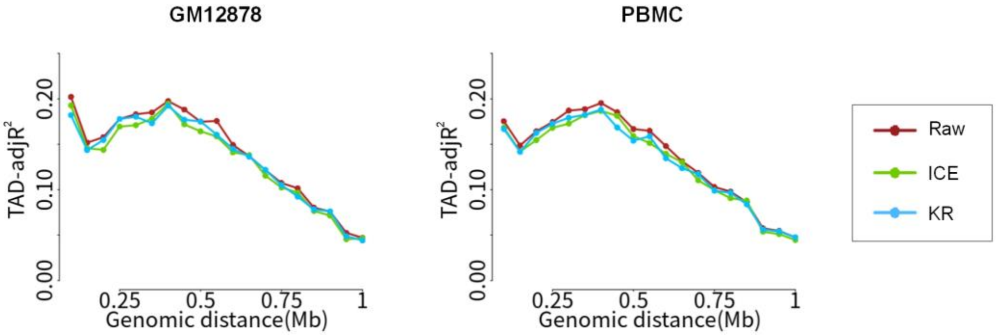

Supplementary Fig. S13. Accuracy of domain assembly on single-cell Hi-C data with different normalization preprocessings. The TAD-like domains were called by scKTLD on single-cell Hi-C contact matrices for chromosome 1 of 17 GM12878 cells and 18 PBMC cells from Tan's dataset at 50 kb resolution.

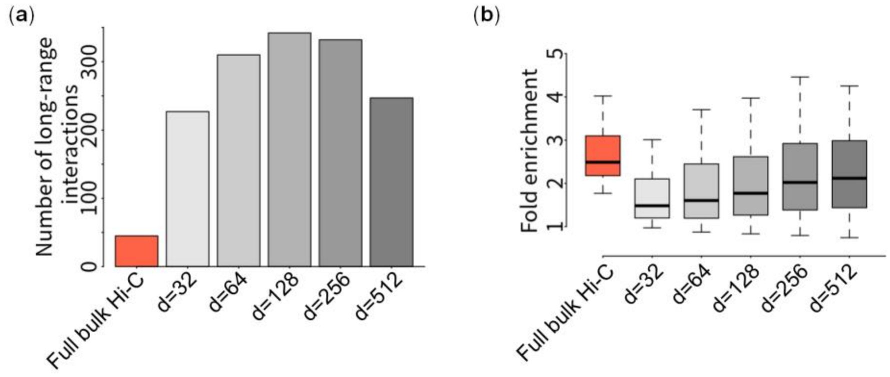

Supplementary Fig. S14. The comparison of the frequency and intensity of long-range interactions called on the full bulk Hi-C contact matrix and reconstructed Hi-C contact matrices with different embedding dimensions. (a) The number of long-range interactions. (b) The fold enrichment score of long-range interactions from the donut background. The long-range interactions were called by HICCUPS on the full bulk Hi-C contact matrix and reconstructed Hi-C contact matrices for chromosome 1 of GM12878 cell line in Rao's dataset at 50 kb resolution.

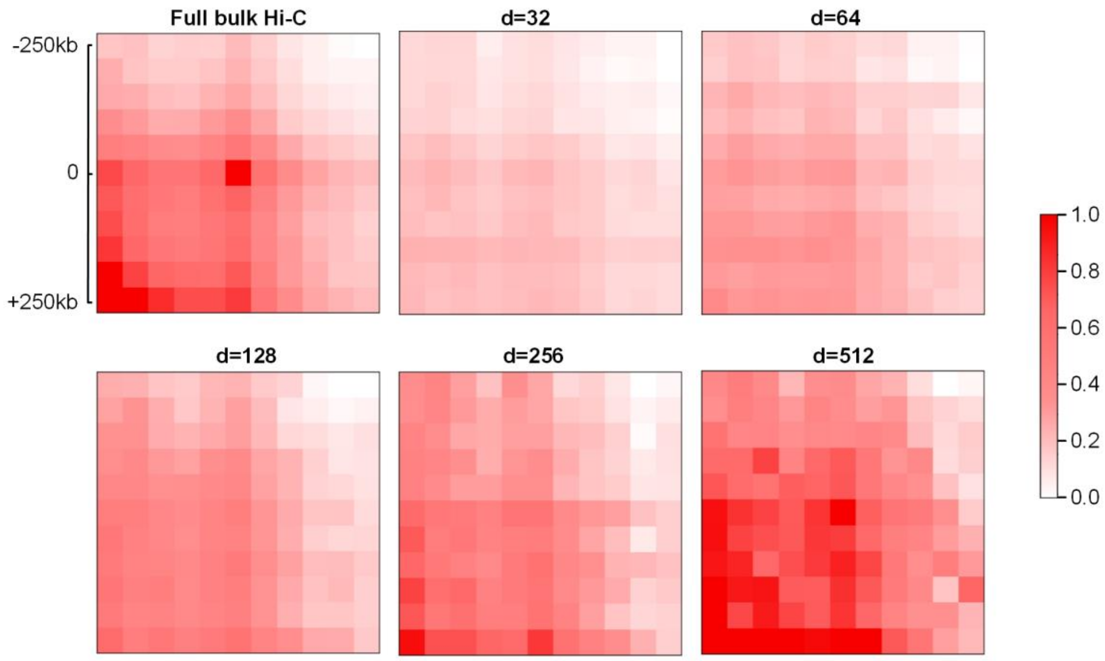

Supplementary Fig. S15. The comparison of APA plots for the long-range interactions called on the full bulk Hi-C contact matrix. The long-range interactions were called by HICCUPS on the full bulk Hi-C contact matrix for chromosome 1 of GM12878 cell line in Rao's dataset at 50 kb resolution, and were visualized by APA on the full bulk Hi-C contact matrix and the reconstructed ones with different embedding dimensions. The APA was performed by averaging all the submatrices surrounding each long-range interaction in a contact matrix.

III. Supplementary Tables

Supplementary Table S1. Experimental bulk Hi-C data involved in this paper

Cell line	Replicate	Resolution
GM12878	GSM1551550_HIC001	50 kb /25 kb
GM12878	GSM1551551_HIC002	50 kb /25 kb
GM12878	GSM1551552_HIC003	50 kb /25 kb
GM12878	GSM1551553_HIC004	50 kb /25 kb
GM12878	GSM1551554_HIC005	50 kb /25 kb
GM12878	GSM1551585_HIC036	50 kb /25 kb
GM12878	GSM1551586_HIC037	50 kb /25 kb
GM12878	GSM1551589_HIC040	50 kb /25 kb
GM12878	GSM1551590_HIC041	50 kb /25 kb
GM12878	GSM1551591_HIC042	50 kb /25 kb
K562	GSM1551619_HIC070	10 kb

Supplementary Table S2. Simulated Hi-C data involved in this paper

Chromosome segment (Simulated cell type)		Distance threshold	Number of matrices	Resolution
chr 8: 25M-30M	Single-cell Hi-C	500	100	50 kb
		750	100	50 kb
		1000	100	50 kb
	Reference	NA	100	50 kb
chr 8: 100M-105M	Single-cell Hi-C	500	100	50 kb
		750	100	50 kb
		1000	100	50 kb
	Reference	NA	100	50 kb

Supplementary Table S3. Experimental single Hi-C data involved in this paper

Datasets	Cell types	Number of cells	Resolution	Accession
Tan’s	GM12878	18	50 kb	GSE80006
	PBMC	17	50 kb	
Flyamer’s	ZygM	22	50 kb	GSE117876
	ZygP	18	50 kb	
	Oocyte	70	50 kb	

Supplementary Table S4. ChIP-seq data involved in this paper

Cell line	Type	Accession
GM12878	CTCF	ENCFF002UKL
GM12878	Rad21	ENCFF820XGC
GM12878	Smc3	ENCFF380XGR
GM12878	H3K4me3	ENCFF711AYD

Supplementary Table S5. The runtime and memory consumption on Tan's and Flyamer's dataset at 50 kb resolution

Methods	Runtime		Memory consumption	
	Tan's dataset	Flyamer's dataset	Tan's dataset	Flyamer's dataset
**scKTLD**	49.6 min	155.4 min	0.6 GB	0.6 GB
deDoc	82.8 min	275.7 min	2.4 GB	2.4 GB
TopDom	43.2 min	161.2 min	0.4 GB	0.4 GB
SpectralTAD	51.5 min	166.2 min	0.5 GB	0.5 GB
GRiNCH	3073.9 min	10153.2 min	1.3 GB	1.3 GB
**Higashi**	141.3 min	407.5 min	10.7 GB	18.1 GB
**scHiCluster(4)**	277.9 min	652.7 min	1.2 GB×4	2.8 GB×4
**scHiCluster(8)**	203.5 min	532.7 min	1.2 GB×8	2.8 GB×8
**scHiCluster(12)**	163.8 min	407.5 min	1.2 GB×12	2.8 GB×12
**deTOKI(4)**	214.3 min	687.9 min	0.4 GB×4	0.4 GB×4
**deTOKI(8)**	156.9 min	528.2 min	0.4 GB×8	0.4 GB×8
**deTOKI(12)**	126.3 min	417.7 min	0.4 GB×12	0.4 GB×12

The bolded indicate methods specifically developed for single-cell Hi-C data, and the numbers inside the parentheses behind scHiCluster and deTOKI indicate the numbers of threads.

IV. References

Bau, D. and Marti-Renom, M.A. Genome structure determination via 3C-based data integration by the Integrative Modeling Platform. *Methods* 2012;58(3):300-306.

Killick, R., Fearnhead, P. and Eckley, I.A. Optimal detection of changepoints with a linear computational cost. *Journal of the American Statistical Association* 2012;107(500):1590-1598.

Kipf, T.N. and Welling, M. Semi-supervised classification with graph convolutional networks. *arXiv preprint arXiv:1609.02907* 2016.

Rao, S.S., *et al.* A 3D map of the human genome at kilobase resolution reveals principles of chromatin looping. *Cell* 2014;159(7):1665–1680.

Serra, F., *et al.* Automatic analysis and 3D-modelling of Hi-C data using TADbit reveals structural features of the fly chromatin colors. *PLoS Comput. Biol.* 2017;13(7):e1005665.

Tan, L., *et al.* Three-dimensional genome structures of single diploid human cells. *Science* 2018;361(6405):924-928.

Zhang, J., *et al.* ProNE: Fast and Scalable Network Representation Learning. In, *IJCAI*. 2019. p. 4278-4284.
